# The Ly6g^high^ Neutrophil Subset Dictates Breast Cancer Lung Metastasis via CD8^+^ T Cell Death

**DOI:** 10.34133/cancomm.0003

**Published:** 2026-01-27

**Authors:** Rui Wang, Xiaoqi Liu, Yixuan Hou, Shanchun Chen, Yongcan Liu, Zexiu Lu, Chao Chang, Die Meng, Jing Chen, Xiaojiang Cui, Zhengrong Shi, Xueying Wan, Manran Liu

**Affiliations:** ^1^ Key Laboratory of Laboratory Medical Diagnostics, Chinese Ministry of Education, Chongqing Medical University, Chongqing 400016, P. R. China.; ^2^ Western Institute of Digital-Intelligent Medicine, Chongqing 401329, P. R. China.; ^3^ Pediatric Research Institute, Children’s Hospital of Chongqing Medical University, National Clinical Research Center for Child Health and Disorders, Ministry of Education Key Laboratory of Child Development and Disorders, Chongqing Key Laboratory of Pediatrics, Chongqing 400014, P. R. China.; ^4^ Experimental Teaching Center of Basic Medicine Science, Chongqing Medical University, Chongqing 400016, P. R. China.; ^5^ Department of Surgery, Department of Obstetrics and Gynecology, Samuel Oschin Comprehensive Cancer Institute, Cedars-Sinai Medical Center, Los Angeles, CA 91006, USA.; ^6^ Department of Hepatobiliary Surgery, the First Affiliated Hospital of Chongqing Medical University, Chongqing 400016, P. R. China.

## Abstract

**Background:** Lung metastasis is a leading cause of breast cancer (BC)-related mortality, driven by the immunosuppressive traits of the metastatic tumor microenvironment. However, the mechanisms underlying cell–cell crosstalk in shaping immune evasion within the metastatic niche remain poorly defined. Neutrophil extracellular traps (NETs) and their associated proteins, such as cathelicidin, have emerged as key mediators of metastatic regulation in cancer. Here, we aimed to decipher the interaction between a neutrophil subset characterized by high expression of lymphocyte antigen 6 complex locus g (Ly6g^high^) and cluster of differentiation 8-positive T lymphocytes (CD8^+^ T cells), mediated via cathelicidin embedded in NETs, as well as their synergistic mechanism and cooperative role in promoting lung metastasis of BC. **Methods:** We characterized neutrophil heterogeneity and functional dynamics by performing single-cell RNA sequencing and flow cytometry on lung tissues derived from murine models of BC lung metastasis. We utilized cathelicidin-related antimicrobial peptide (*Cramp*) knockout mice to dissect the role of cathelicidin in NETs. The spatial colocalization of apoptotic CD8^+^ T cells and NETs was analyzed using multiplex immunofluorescence, and the molecular interactions were probed by protein binding assays. **Results:** Neutrophils in the lung metastatic niche were classified into 2 subsets based on the Ly6g expression: Ly6g^high^ and Ly6g^low^ neutrophils. Ly6g^low^ neutrophils, which were recruited in the macrometastatic stage, exhibited myeloid-derived suppressor cell-like characteristics. Notably, Ly6g^high^ neutrophils induced CD8^+^ T cell apoptosis through NET formation, with apoptotic CD8^+^ T cells spatially clustered within NET-rich areas. Mechanistically, NET-derived cathelicidin (Cramp in mice) directly bound to mitochondrial adenine nucleotide translocator 1 (Ant1) in CD8^+^ T cells, triggering conformational changes and complex formation with voltage-dependent anion channel 1 (Vdac1). These events resulted in the opening of the mitochondrial permeability transition pore and loss of mitochondrial membrane potential. **Conclusions:** Our study demonstrates that Ly6g^high^ neutrophils play a critical role in immunosuppression and immune evasion through NET-induced apoptosis of CD8^+^ T cells. These findings underscore the importance of NETs and cathelicidin in BC lung metastasis, suggesting their potential as therapeutic targets in restoring antitumor immunity and in preventing metastatic progression.

## Background

Tumor metastasis to distant organs is responsible for the majority of cancer-related deaths [1]. The metastatic tumor microenvironment (mTME), like the tumor microenvironment (TME) in the primary tumor, is a complex and unique tumor-surrounding ecosystem. It is composed of cancer cells and diverse nonmalignant cell types, including immune cells and stromal cells, and exhibits site-specific heterogeneity across different distant organs [2,3]. The unique tissue-resident cell types and recruited immune cells (e.g., neutrophils, macrophages, and T cells) in mTME play a crucial and context-dependent role in regulating the fate of disseminated tumor cells (DTCs) [4,5]. Moreover, the dynamic changes of immune cells in metastatic tumors shape the behavior of DTCs and their ability to evade immune surveillance [4]. Tumor-associated macrophages (TAMs), for example, promote DTC dormancy, while neutrophils and monocytes can reactivate dormant DTCs over time with the immune landscape of the mTME shifting [6,7]. Therefore, the systematic mapping of the immune landscape and elucidation of cell–cell interactions in mTME will advance understanding of metastasis formation.

Neutrophils have long been regarded as merely playing a bystander role in cancer development. However, recent evidence has implicated that neutrophils act as a key player in tumor metastasis [8]. Some studies propose that neutrophil accumulation in distant organs contributes to DTCs’ extravasation and proliferation by up-regulating prometastatic proteins [9]. On the other hand, neutrophils are involved in regulating metastasis via neutrophil extracellular traps (NETs), which are the web-like structures composed of DNA, histones, granule proteins, and antimicrobial peptides, including neutrophil elastase (Ne), cathepsin g, and cathelicidin [10]. It is proposed that NETs may facilitate the formation of metastatic lesions through site-specific mechanisms [11]. Nevertheless, the role of accumulated neutrophils, particularly whether a distinct subset of neutrophils in distant lung metastatic tissues, and how they remodel the TME remain unknown.

CD8^+^ cytotoxic T cells (CTLs) are pivotal immune effector cells within the TME. It is well established that their functional impairment or numerical decline contributes to immunosuppression and disease progression in metastatic tumors [12,13]. Previous studies have suggested that mitochondrial dysfunction may be a potentially critical cause of CD8^+^ T cell exhaustion. This is evidenced by findings that CD8^+^ T cells from cancer patients and tumor-bearing mice exhibit decreased mitochondrial mass and impaired function [14]. Furthermore, abnormal degradation of mitochondria-associated mRNAs can disrupt the mitochondrial membrane potential (ΔΨm), thereby compromising their antitumor activity [15]. Beyond intrinsic T cell defects, recent advancements have deepened our understanding of different cells in the TME, such as cancer cells and myeloid cells, contributing to CD8^+^ T cell exhaustion [16]. In contrast, the role of neutrophils, specifically the mechanism by which accumulated neutrophil subsets disrupt mitochondrial function in CD8^+^ T cells, ultimately leading to functional impairment and reduced abundance in metastatic foci, remains poorly understood.

To delineate the immunosuppressive landscape of the lung metastatic microenvironment during breast cancer (BC) lung metastasis, we performed single-cell RNA sequencing (scRNA-seq) of murine lung tissues at different metastatic stages to provide a characterization of immune cell populations. This study aimed to define the cellular interactions that drive immune evasion during BC lung metastasis, with a particular focus on neutrophil heterogeneity and its functional impact on CD8^+^ T cells. Our work sought to establish a comprehensive view of how specific neutrophil subsets shape the premetastatic niche and foster an immunosuppressive milieu, thereby offering insight into potential therapeutic strategies for BC with lung metastasis.

## Materials and Methods

### Clinical samples

Peripheral venous blood samples were collected from 160 BC patients and 50 healthy volunteers. Primary tumor tissues and matched lung metastatic lesions were collected from an additional 6 BC patients. All patients were recruited from the First Affiliated Hospital of Chongqing Medical University (Chongqing, China) between January 2019 and January 2025, and written informed consent was obtained from all participants. The Institutional Ethics Committee of Chongqing Medical University approved the study (approval number: 2021084), per the Declaration of Helsinki. All samples were anonymized, and analyses were performed in a blinded manner. Detailed clinical information of all patients and healthy volunteers is listed in Tables S1 and S2. Correlation data between peptidyl arginine deiminase 4 (PADI4) and interleukin 1β (IL1B), C-X-C motif chemokine ligand 2 (CXCL2), as well as C-C motif chemokine ligand 23 (CCL23) in human normal lung tissues were derived from the TNMplot database (https://tnmplot.com).

### Cell culture

The American Type Culture Collection (ATCC; Manassas, VA, USA) provided the mouse BC cell lines 4T1 and E0771. These cells were cultured in RPMI 1640 medium, supplemented with 10% fetal bovine serum (FBS; AUS-01S-02, Cell-Box, Changsha, Hunan, China) and 1% penicillin–streptomycin (C0222, Beyotime, Shanghai, China). They were maintained at 37 °C in an environment with 5% CO₂ and high humidity. To track bioluminescence, breast tumor cells (4T1 and E0771) were infected with a fusion protein reporter that included firefly luciferase (Luc) using retroviral methods. These cell lines were named Luc-4T1 and Luc-E0771, respectively. All cell lines were tested for mycoplasma contamination with polymerase chain reaction (PCR)-based tests and confirmed through short tandem repeat (STR) profiling before being used.

### Mouse

Female C57BL/6 mice (6 to 8 weeks old) and BALB/c (6 to 8 weeks old) were purchased from the Laboratory Animal Center of Chongqing Medical University (Chongqing, China). S. Liu (Shanghai Cancer Center and Institutes of Biomedical, Fudan University, Shanghai, China) provided the murine mammary tumor virus (MMTV)–polyoma virus middle antigen (PyMT) mice (on C57BL/6 background) as a gift. Cathelicidin-related antimicrobial peptide (*Cramp*) knockout (C57BL/6J-*Cramp*^em1C^/Cya, hereafter referred to as *Cramp*^−*/*−^) mice were purchased from Cyagen Bioscience (Suzhou, Jiangsu, China). All animals were maintained under specific pathogen-free (SPF) conditions in a temperature-controlled (22 ± 2 °C) environment with a 12-h light/dark cycle and were allowed free access to standard rodent chow and water. All animal experiments were approved by the Animal Ethics Committee of Chongqing Medical University (approval number: 2021084) and performed in accordance with the Guide for the Care and Use of Laboratory Animals published by the National Institutes of Health [7].

Given the pulmonary tropism of 4T1 and E0771 BC cell lines, BALB/c or C57BL/6 mice were injected with mycoplasma-free Luc-4T1 cells [1 × 10^6^ cells in 100 μl of phosphate-buffered saline (PBS)] or Luc-E0771 cells (5 × 10^5^ cells in 100 μl of PBS) at the fourth mammary fat pad. To observe metastasis, mice were injected with d-luciferin (100 mg/kg, 40901ES03, Yeasen, Shanghai, China), followed by bioluminescence imaging with the in vivo imaging system (IVIS) LB 983 system (Berthold, Stuttgart, Germany). Lungs with metastatic signal were harvested and dissociated by collagenase type I (1904MG100, BioFroxx, Lower Saxony, Germany) and then inoculated in complete medium to obtain DTCs. DTCs were purified and reinjected into the fourth mammary fat pad for the next round of selection. In summary, tumor cells with enhanced organotropic metastasis ability (4T1-LM3 and E0771-LM3; LM refers to lung metastasis) were obtained after 3 rounds of in vivo selection. Mice receiving orthotopic injections of 4T1-LM3 or E0771-LM3 cells were categorized into 3 stages of metastasis: the premetastatic stage, defined as the absence of visible tumor nodules in lung tissues; the micrometastatic stage, defined as the presence of nodules with a longest diameter ≤300 μm; and the macrometastatic stage, defined as the presence of nodules with a longest diameter >300 μm.

To inhibit NET formation, 4T1-LM3 (1 × 10^6^ cells in 100 μl of PBS) and E0771-LM3 (5 × 10^5^ cells in 100 μl of PBS) were injected into the fourth mammary fat pad of mice. The PADI4 inhibitor GSK484 (20 mg/kg, HY-100514, MedChemExpress, Monmouth Junction, NJ, USA) was administered intraperitoneally daily, starting either on the day of tumor cell injection or at the pre-, micro-, or macrometastatic stage. Primary tumors were surgically resected at week 3 post-inoculation, followed by intraperitoneal injection of either interleukin-1β (Il-1β) antibody (200 μg per mouse, BE0246, BioXCell, West Lebanon, NH, USA) or immunoglobulin G (IgG) isotype control antibody (200 μg per mouse; BE0091, BioXCell) 3 times weekly until the macrometastatic stage.

To induce NET formation, primary tumors were resected on day 21 after the orthotopic injection of 4T1-LM3 cells, followed by daily intraperitoneal injections of recombinant Il-1β (rIl-1β; 8 ng per mouse, HY-P7073, MedChemExpress) until the macrometastatic stage. At the macrometastatic stage, all mice were euthanized, and the lung tissues were collected for further evaluation.

To deplete macrophages, primary tumors of BALB/c mice were surgically resected at week 3 post-orthotopic inoculation. This was coupled with thrice-weekly intravenous administration of F4/80 antibody (150 μg per mouse, BE0206, BioXCell) or IgG control (150 μg per mouse, BE0090, BioXCell) until macrometastatic stage.

To assess CD8^+^ T cell infiltration in the lung mTME, mice were treated daily with the PADI4 inhibitor GSK484 (20 mg/kg) and administered intranasally with either Cramp (30 mg/kg, 376364-36-2, Go Top Peptide Biotech, Hangzhou, Zhejiang, China) or bongkrekic acid (BKA; 200 μg/kg, HY-136406, MedChemExpress) 3 times per week after E0771-LM3 cell injection, continuing to the macrometastatic stage.

To inhibit NET formation and enhance immunotherapy, mice received administration of the PADI4 inhibitor GSK484 (20 mg/kg) daily, beginning at tumor cell inoculation. During the micrometastatic stage, the primary tumors were resected, followed by intraperitoneal injection of either anti-PD-1 monoclonal antibody (200 μg per mouse, BE0146, BioXCell) or IgG isotype control (200 μg per mouse, BE0089, BioXCell) every 3 d until the macrometastatic stage.

To minimize animal suffering, all surgical procedures were performed under anesthesia with 1.5% isoflurane inhalation. Humane endpoints were defined as weakness or a 20% loss in body weight compared to the control littermates. Animals meeting these criteria were euthanized by cervical dislocation. At the end of the experiment, tissues were collected for further analysis under institutional biosafety protocols.

### Isolation of neutrophils

Bone marrow-derived neutrophils were harvested from BALB/c and C57BL/6 mice. In sterile Hanks’ buffered salt solution (HBSS; BL559A, Biosharp, Hefei, Anhui, China), the femurs and tibias were separated to extract the bone marrow. Bone marrow cells were flushed using HBSS buffer and centrifuged (400*g*, 5 min, 4 °C). The cell pellets were resuspended on ice with red blood cell (RBC) lysis buffer (BL503A, Biosharp) and washed twice using HBSS buffer. Neutrophils were subsequently isolated using a Mouse Bone Marrow Neutrophil Isolation Kit (TBD2013NM, TBD Sciences, Tianjin, China).

Lung-derived neutrophils were primarily isolated from BALB/c mice or C57BL/6 mice. Lungs were dissected, mechanically dissociated, and then digested with collagenase type I (30 min, 37 °C). The resulting cell suspension was filtered through a 70-μm nylon cell strainer with RPMI 1640 medium and centrifuged (500*g*, 5 min, 4 °C). Cells were treated with RBC lysis buffer, centrifuged (400*g*, 5 min, 4 °C), washed in HBSS, and recentrifuged under identical conditions. Cells were stained with fluorescein isothiocyanate (FITC) anti-mouse CD45 antibody (157214, BioLegend, San Diego, CA, USA), peridinin–chlorophyll–protein complex/Cyanine5.5 (PerCP/Cyanine5.5) anti-mouse CD11b antibody (101228, BioLegend), and phycoerythrin (PE) anti-mouse Ly6g antibody (127608, BioLegend) in the dark (15 min, 4 °C). Cells were then washed twice in HBSS buffer (400*g*, 5 min, 4 °C) and sorted using the BD FACSCanto Plus Flow Cytometer (BD Biosciences, Franklin Lakes, NJ, USA).

Peripheral blood donated by healthy volunteers and BC patients was used for human neutrophil isolation. RBCs were lysed using RBC lysis buffer and washed with HBSS buffer (400*g*, 5 min, 4 °C). The remaining cells were stained with FITC anti-human CD45 antibody (982316, BioLegend), allophycocyanin (APC) anti-human CD66b antibody (396906, BioLegend), and PE anti-human CD84 antibody (326008, BioLegend) in the dark (30 min, 4 °C). After twice washing with HBSS buffer (400*g*, 5 min, 4 °C), CD45^+^CD66b^+^CD84^high^ cells or CD45^+^CD66b^+^CD84^low^ cells were isolated by BD FACSCanto Plus Flow Cytometer. All the neutrophils were resuspended in serum-free Dulbecco’s modified Eagle’s medium (DMEM) for subsequent experiments.

### Extraction and visualization of NETs

Neutrophils were cultured in 24-well plates with serum-free DMEM followed by overnight activation with phorbol 12-myristate 13-acetate (PMA; 200 nM, 16561-29-8, Sigma-Aldrich, St. Louis, MO, USA) to induce NET formation. After discarding the supernatant, adherent NETs were collected by rinsing the well bottom with 2 ml of cold PBS, followed by centrifugation (1,000*g*, 10 min, 4 °C). DNA concentrations in NETs were measured using a NanoDrop Spectrophotometer (Thermo Fisher Scientific, Waltham, MA, USA), and the NETs were then used for subsequent experiments.

To assess NET formation by neutrophil subsets, we isolated neutrophils from 2 sources: lung tissues of tumor-bearing mice and peripheral blood of BC patients using flow cytometry. The sorted neutrophils were then seeded onto poly-l-lysine-coated slides pre-placed in 24-well plates. Cells were stimulated with PMA (200 nM), Il-1β (1 ng/ml, 90140ES10, Yeasen), Cxcl2 (100 ng/ml, 250-15, Proteintech, Chicago, IL, USA), or Ccl6 (100 ng/ml, HY-P7143, MedChemExpress) for 6 h under static conditions at 37 °C. Post-stimulation, NET formation was quantified via immunofluorescence staining. Briefly, cells were fixed with 4% paraformaldehyde (PFA), permeabilized with 0.5% Triton X-100 (P0096, Beyotime) for 10 min, and blocked with 10% goat serum for 30 min at room temperature. Subsequently, samples were incubated overnight at 4 °C with the following antibodies: rat anti-mouse Ly6g (1:200; 27601, BioLegend) and rabbit anti-histone H3 (1:200; ab5103, Abcam, Cambridge, MA, USA). After incubation with fluorescently labeled secondary antibodies and 4′,6-diamidino-2-phenylindole (DAPI) (1 μg/ml), NET structures were visualized using a DMi8 confocal microscope (Leica, Wetzlar, Germany). Quantification of all fluorescent images was performed by ImageJ software (version 1.8.0; https://imagej.net/software).

### Quantification of myeloperoxidase–DNA

As described previously, myeloperoxidase (MPO)–DNA complexes in murine and human plasma were quantified by capture enzyme-linked immunosorbent assay (ELISA) to determine NET levels [11]. An anti-MPO polyclonal antibody (PA5-16672, Thermo Fisher Scientific), diluted to 5 μg/ml with carbonate buffer (pH 9.6, CB01100, Thermo Fisher Scientific), was used as the capture antibody to coat 96-well plates overnight at 4 °C. After blocking with 1% bovine serum albumin (BSA), 100 μl of plasma from mice or BC patients, or the standards (EK1133S, human; EK2133S, mouse, Multi science, Hangzhou, Zhejiang, China) were added to the plates, the plates were incubated with peroxidase-labeled anti-DNA monoclonal antibody (11774425001, Roche, Basel, Switzerland) for 1 h at 37 °C. After washing with PBS 3 times, the peroxidase substrate 3,3′,5,5′-tetramethylbenzidine (TMB; T0440, Sigma-Aldrich) was added and incubated for 40 min at 4 °C. Optical density was measured at 405 nm using a microplate reader (Multiskan GO, Thermo Fisher Scientific). Standard curves were used to validate assay performance.

### Coculture of neutrophils and CD8^+^ T cells

Flat-bottom 48-well plates were coated with anti-CD3ε antibody (3 μg/ml, 550275, BD Biosciences) diluted in PBS (1:10, 2 h, 37 °C). The plates were then washed once with PBS. To isolate CD8^+^ T cells, spleens were harvested from mice, and CD8^+^ T cells were purified using the CD8a^+^ T Cell Isolation Kit (130-117-044, Miltenyi Biotec, Bergisch Gladbach, Germany). CD8^+^ T cells were then resuspended in complete RPMI 1640 medium supplemented with 10% FBS, 1% sodium pyruvate, 1% glutamine, and 0.1% mercaptoethanol with recombinant mouse IL-2 (100 U/ml, CK24, Novoprotein, Suzhou, Jiangsu, China) and anti-CD28 antibodies (1 μg/ml, 553294, BD Biosciences). For the proliferation assay, the purified CD8^+^ T cells were labeled with 2 μM carboxyfluorescein succinimidyl ester (CFSE; C34554, Invitrogen, Carlsbad, CA, USA). The labeled CD8^+^ T cells were cocultured with Ly6g^high^ or Ly6g^low^ neutrophils isolated by flow cytometry at a 1:1 ratio. After 3 d of coculture, proliferation and function of CD8^+^ T cells were analyzed using a BD FACSCanto Plus Flow Cytometer and FlowJo software (version 10.0; https://www.flowjo.com).

### Cell viability assay

CD8^+^ T cells (5 × 10^4^ cells) were treated with neutrophil-derived conditioned medium (CM-Neu), NETs (5 μg/ml), or NETs (10 μg/ml) in 96-well plates. Following 24-h culture, cells were treated with CellTiter-Blue Reagent (4 h, 37 °C; G8090, Promega, Madison, WI, USA). Viable cells were quantified with a Varioskan LUX multimode reader (Thermo Fisher Scientific).

### Flow cytometry

To detect or isolate distinct cell populations within murine lung tissue, lungs were dissected, mechanically dissociated, and then digested with collagenase type I (30 min, 37 °C). The resulting cell suspension was filtered through a 70-μm nylon cell strainer with RPMI 1640 medium and centrifuged (500*g*, 5 min, 4 °C). The cell pellets were resuspended in RBC lysis buffer and centrifuged (400*g*, 5 min, 4 °C), followed by washing with HBSS buffer. Then, the cells were stained with the respective antibodies in the dark (15 min, 4 °C). Stained cells were analyzed and sorted using a BD FACSCanto Plus Flow Cytometer, and data were processed with FlowJo software (version 10.0). Neutrophils (Ly6g^+^), macrophages (F4/80^+^), basophils (CD200R3^+^), and natural killer (NK) cells (Nkp46^+^) were gated from CD45^+^CD11b^+^ cells; CD8^+^ and CD4^+^ T cells were gated from CD45^+^CD3^+^ cells; B cells (CD19^+^) and dendritic cells (CD11c^+^MHC-II^+^) were gated from the CD45^+^ population. CD326^−^CD45^−^CD31^−^ cells were defined as fibroblasts, and CD326^−^CD45^−^CD31^+^ cells were regarded as endothelial cells. To define and quantify the Ly6g^high^ and Ly6g^low^ neutrophil subsets in mice and the CD84^high^ and CD84^low^ subsets in humans, we first established the positive gating threshold using fluorescence-minus-one (FMO) controls. Subsequently, the boundary between “high” and “low” subsets was determined based on a clear inflection point observed in the fluorescence intensity histogram. The antibodies for cell subtype gating are listed in Table S3.

To assess cytokine production of CD8^+^ T cells, cells were stimulated with PMA (50 ng/ml) and ionomycin (500 ng/ml, 50401ES03, Yeasen), and then treated with the Golgi transport inhibitor monensin (554724, BD Biosciences) for 4 h at 37 °C. Cells were collected, fixed, and permeabilized using Cytofix/Cytoperm Kit (554715, BD Biosciences), followed by staining with PE anti-mouse interferon-γ (IFN-γ) antibody (505807, BioLegend) in the dark (40 min, 4 °C). Subsequently, cells were analyzed using a BD FACSCanto Plus Flow Cytometer and FlowJo software (version 10.0).

To assess CD8^+^ T cell apoptosis, in vitro experiments were performed as follows: CD8^+^ T cells were washed twice with HBSS, incubated with Annexin V and propidium iodide (PI) reagent (40302ES60, Yeasen) in the dark (15 min, 4 °C), and immediately analyzed by flow cytometry. Separately, for in vivo analysis, single-cell suspensions from murine lung tissues were prepared. Cells were stained with APC anti-mouse CD3 antibody, PerCP/Cyanine5.5 anti-mouse CD8, and Zombie Aqua Fixable Viability Kit (423101, BioLegend). All stained cells were analyzed using a BD FACSCanto Plus Flow Cytometer and FlowJo software (version 10.0).

To investigate the potential of macrophages to induce reactive oxygen species (ROS) in neutrophil subsets, we isolated macrophages from the macrometastatic lung tissue of BALB/c mice. Briefly, the macrophages (5 × 10^5^ cells) were cultured for 24 h to generate macrophage-derived conditioned medium (CM-MΦ). We then treated Ly6g^high^ and Ly6g^low^ neutrophils with this CM-MΦ, in the presence or absence of an Il-1β neutralizing antibody (5 μg/ml, BE0246, BioXCell), for 24 h. After treatment, the cells were incubated with the ROS-sensitive probe 2′,7′-dichlorodihydrofluorescein diacetate (DCFH-DA; 10 μM, 50101ES01, Yeasen) at 37 °C for 20 min, washed twice with PBS, and analyzed immediately using a BD FACSCanto Plus flow cytometer. Data were analyzed with FlowJo software (v10.0).

### Immunofluorescence staining

Mouse lung tissues were fixed in 4% PFA at 4 °C overnight and then paraffin-embedded and sectioned into 4-μm-thick slices. The sections underwent deparaffinization, rehydration, and permeabilization, followed by a citric acid antigen retrieval process that took place at high temperature and pressure (100 °C and 70 to 80 kPa) for 15 min. Once naturally cooled, the slides were blocked and incubated with primary antibodies (12 h, 4 °C), ultrasensitive horseradish peroxidase (HRP)-conjugated goat anti-mouse/rabbit IgG polymer (50 min, 25 °C; AFIHC025, Aifang Biotechnology, Changsha, Hunan, China), and Opal TSA dyes (10 min, 25 °C; AFIHC025, Aifang Biotechnology). Subsequent staining cycles involved repeating the antigen retrieval step (15 min, 100 °C) followed by incubation with primary antibodies (12 h, 4 °C), ultrasensitive HRP-conjugated goat anti-mouse/rabbit IgG polymer (50 min, 25 °C), and Opal TSA dyes (10 min, 25 °C). Finally, the sections were stained with DAPI (ServiceBio, Wuhan, Hubei, China). The antibodies used for immunofluorescence staining are listed in Table S3. Images were captured using OLYMPUS cellSens standard software (version 1.11, OLYMPUS, Tokyo, Japan) and analyzed by Case Viewer software (version 2.3; https://www.3dhistech.com/).

### Immunohistochemical staining of tissue samples

Mouse lung tissues were fixed in 4% PFA overnight at 4 °C for paraffin-embedding, washed with PBS, and transferred into 70% ethanol. The tissues were embedded in paraffin and sectioned into 4-μm-thick slices. Lung sections were deparaffinized, rehydrated, and permeabilized. Antigen retrieval was performed using 10 mM sodium citrate buffer (pH 6.0). This process took place at a high temperature and pressure (100 °C and 70 to 80 kPa) for 15 min, followed by natural cooling to room temperature to preserve tissue architecture. The sections were incubated with primary antibodies at 4 °C overnight. Subsequently, the sections were exposed to secondary antibodies at room temperature for 1 h. Staining was performed using diaminobenzidine (DAB) for 2 min. Subsequently, the slices were stained with hematoxylin for 30 s and mounted with a resin-based mounting medium to ensure permanent preservation and optical clarity for subsequent microscopic examination. The slides were imaged using an Eclipse 80i Microscope (Nikon, Tokyo, Japan). The antibodies used for immunohistochemical (IHC) staining are listed in Table S3. IHC-stained tissue section images were analyzed using ImageJ software (version 1.8.0). Staining intensity was quantified using integrated optical density (IOD; calculated as area × mean gray value using ImageJ, Analyze > Measure) and H-score (assigned using the IHC Profiler plugin in ImageJ, classifying staining intensity to 4 tiers: 0, 1+, 2+, and 3+). For data normalization, IOD values underwent background subtraction of negative controls followed by normalization relative to positive controls.

### Giemsa stain

Neutrophil smears were fixed by immersion in absolute methanol for 3 min. The fixed and air-dried smears were then placed in Coplin jars containing freshly prepared Giemsa working solution, ensuring complete immersion of the slides. Staining was performed at room temperature for 15 min. Subsequently, the slides were gently rinsed by directing a slow stream of distilled water or PBS across the slide surface and allowed to dry completely. Initial examination of staining quality and cell distribution was carried out using low-power fields, followed by detailed observation and differential counting under oil immersion.

### Detection of cytosolic ROS and mitochondrial ROS

CD8^+^ T cells were incubated with ROS-sensitive probe DCFH-DA (10 μM, 50101ES01, Yeasen) or MitoSOX red mitochondrial superoxide indicator (5 μM, 40778ES50, Yeasen) in the dark (20 min, 37 °C). Cells were then washed with PBS and stained with Hoechst 33342 (1 mg/ml, Beyotime). Fluorescent signals were analyzed by Leica DMi8 confocal microscopy.

### Colocalization of cathelicidin and mitochondria

CD8^+^ T cells were cocultured with purified NETs on coverslips for 2 h at 37 °C and subsequently stained with a fluorescent mitochondria-specific dye, MitoTracker Red (C1049B, Beyotime), according to the manufacturer’s instructions. Cathelicidin expression in CD8^+^ T cells was then detected by immunofluorescence staining using rabbit anti-cathelicidin antibody (1:100, 12009-1-AP, Proteintech), and fluorescent signals were captured using Leica DMi8 confocal microscopy.

### ΔΨm assay

ΔΨm was assessed using JC-1 fluorescence with a Mitochondrial Membrane Potential Assay Kit (C2006, Beyotime), following the manufacturer’s guidelines. Briefly, CD8^+^ T cells were incubated with JC-1 dye (20 min, 37 °C) and then rinsed 3 times with JC-1 staining buffer. Fluorescent signals were acquired using BD FACSCanto Plus Flow Cytometer and analyzed by FlowJo software (version 10.0).

### Electron microscopy

Initially, CD8^+^ T cells underwent treatment with 3% glutaraldehyde and then with 1% osmium tetroxide. Cells were dehydrated using increasing concentrations of acetone (30%, 50%, 70%, 80%, 90%, and 95%), culminating in 3 treatments with absolute acetone. The samples were then extensively infiltrated with Epon 812 resin for 1 week before being embedded. The ultrathin sections were cut using a diamond knife and then stained with uranyl acetate (10 min) and lead citrate (2 min). Ultrastructural observation was conducted using the JEM-1400-FLASH Transmission Electron Microscope (JEOL, Tokyo, Japan).

### ELISA assay

ELISA kits for Il-1β (MLB00C, R&D Systems, Minneapolis, MN, USA), Cxcl2 (Ek2142, MultiSciences, Hangzhou, Zhejiang, China), and Ccl6 (EM1474, FineTest, Wuhan, Hubei, China) were used to measure the cytokine levels in tissue lysates prepared from normal lungs or lungs at different metastasis stage in mice, following the manufacturer’s instructions. Briefly, lung tissues were washed once with cold PBS, cut into smaller pieces, and homogenized thoroughly in radioimmunoprecipitation assay lysis (RIPA) buffer (P0013C, Beyotime) containing protease inhibitors. The lysates were incubated on ice for 30 min, followed by centrifugation (2,000*g*, 20 min, 4 °C). The protein concentration in the supernatant was measured using the bicinchoninic acid (BCA) Protein Assay Kit (P0009, Beyotime). Protein (1 μg/ml) was used for each ELISA assay.

### Western blotting

For Western blotting, total proteins were extracted from cells using RIPA buffer. The protein concentration was measured using a BCA Protein Assay Kit. Protein (50 μg/ml) was separated by 8% to 12% sodium dodecyl sulfate–polyacrylamide gel electrophoresis (SDS-PAGE) and transferred onto polyvinylidene difluoride (PVDF) membranes (1620184, Bio-Rad, Hercules, CA, USA). Protein transfer was performed at a constant voltage of 100 V, and transfer time was optimized based on target protein size: 60 min for <50 kDa, 90 min for 50 to 100 kDa, and 120 min for >100 kDa proteins. The membranes were blocked with 5% nonfat milk at room temperature for 1 h and subsequently incubated at 4 °C overnight with the primary antibodies. The corresponding antibodies are listed in Table S3. Membranes were then incubated with the appropriate HRP-conjugated secondary antibodies for 2 h at room temperature. Protein bands were detected using the enhanced chemiluminescence system (Bio-Rad), and images were captured using Scion Image software (version 10.0; http://www.scioncorp.com). β-Actin was utilized as an internal control.

### Pull-down assay

For in vitro protein interaction analysis, experiments were performed using a Biotinylated Protein Interaction Pull-Down Kit (21115, Thermo Fisher Scientific) according to the manufacturer’s instructions. Peptides were first labeled with biotin using a Biotinylation Kit (21445, Thermo Fisher Scientific) and then immobilized on streptavidin columns. After the biotinylated peptides were immobilized, the remaining streptavidin binding sites on the columns were blocked with a biotin blocking solution (4 °C, 30 min). The columns were then incubated with recombinant mouse adenine nucleotide translocator 1 (Ant1; R15023m, EIAab, Wuhan, Hubei, China) or recombinant mouse voltage-dependent anion channel 1 (Vdac1; R12942m, EIAab). The columns were washed with washing buffer to reduce nonspecific binding, and elution buffer was added to elute the prey proteins bound to the bait. The eluted proteins were then detected by Western blotting assay.

### Coimmunoprecipitation

For coimmunoprecipitation (Co-IP) experiments, cells were lysed using IP lysis buffer containing protease inhibitor cocktails (P1005, Beyotime). Protein aliquots (500 μg) were precleared by incubation with 20 μl of Dynabeads Protein A (10001D, Invitrogen) for 1 h at 4 °C. The anti-Ant1 antibodies (3 μg per sample, 15997-1-AP, Proteintech) were then added to the precleared samples and incubated overnight at 4 °C. Then, the samples were incubated with 20 μl of Dynabeads Protein A for 2 h at 4 °C. After washing 3 times with cold lysis buffer, the beads were then boiled, and the bound proteins were analyzed by Western blotting. The antibody reagents are listed in Table S3.

### RNA isolation and quantitative real-time PCR

Total RNA was isolated using TRIzol reagent (9109, TaKaRa, Tokyo, Japan). The isolated RNA was then reverse transcribed into cDNA using the PrimeScript RT Reagent Kit (RR047A, TaKaRa). Quantitative real-time PCR (qPCR) was then used to analyze the target gene expression using SYBR Green qPCR Master Mix (HY-K0523, MedChemExpress). Primer sequences are listed in Table S4.

### RNA-mediated interference with Accell small interfering RNA

Isolated CD8^+^ T cells from mice were incubated with Accell delivery mix [Accell small interfering RNA (siRNA) delivery media; B-005000-100, Dharmacon, Waltham, MA, USA] containing 1 μM siRNA and 13.4 U/ml IL-2 for 3 d. Knockdown efficiency was assessed in harvested cells via qPCR prior to functional analyses.

### Bulk RNA sequencing and analysis

For RNA sequencing (RNA-seq) of neutrophil subsets, CD45^+^Ly6g^high^ neutrophils from mouse normal lung tissue and CD45^+^Ly6g^high^ and CD45^+^Ly6g^low^ neutrophils from mouse macrometastatic lung tissue were sorted using a BD FACSCanto Plus Flow Cytometer. For RNA-seq of CD8^+^ T cells, cells were isolated from the spleens of BALB/c mice and then treated with or without NETs (10 μg/ml) for 48 h before collection. Total RNA was extracted using TRIzol reagent, and RNA quality was assessed by a NanoDrop Spectrophotometer (Thermo Fisher Scientific) and an Agilent 2100 Bioanalyzer (Thermo Fisher Scientific). RNA library construction and sequencing were performed by BGI Genomics Co. Ltd. (Shenzhen, Guangdong, China). Differentially expressed gene (DEG) analysis was performed using DESeq2 (version 1.4.5; https://bioconductor.org/packages/release/bioc/html/DESeq2.html) with a significance threshold of *P* value ≤ 0.05 and log_2_ fold change > 0.58. All subsequent analyses were conducted based on the Dr. Tom analysis system (BGI; https://biosys.bgi.com).

### scRNA-seq and analysis

Lungs from healthy mice and those at different metastasis stages were dissociated using the Tumor Dissociation Kit (130-096-730, Miltenyi Biotec). The resulting cell suspensions were then passed through a 70-μm nylon cell strainer, resuspended in an RBC lysis buffer on ice, then washed with HBSS buffer, and centrifuged (400*g*, 5 min, 4 °C). The cell viability was measured using trypan blue staining under a microscope. Cells with a viability of at least 80% were qualified for RNA library construction. Single-cell suspensions were partitioned into gel bead-in-emulsions (GEMs) using the automated Chromium Controller, within which mRNA was reverse transcribed into cDNA; subsequently, GEMs were broken, and cDNA was purified, amplified, and subjected to end repair and adaptor ligation via double-sided solid-phase reversible immobilization (SPRI), followed by PCR amplification to generate final libraries. scRNA-seq was performed using the MGISEQ 2000 platform (BGI).

Cell Ranger (version 3.2.0; https://support.10xgenomics.com) was used to combine the raw data expression matrices produced by each sample. Cells were subjected to quality control based on the quantity of genes detected in each cell. Cells that had fewer than 200 genes detected or cells that had more than 90% of the maximum genes were excluded. Cells were arranged based on their mitochondrial read ratio in a descending order for the mitochondrial measurement, and only the top 15% of cells were excluded. After normalizing the gene expression datasets, a principal components analysis (PCA) with 15 components was performed for dimensionality reduction. The resulting clusters were then visualized in 2 dimensions. Distinctive gene expression patterns were detected in various samples by utilizing the “FindMarkers” function in Seurat (version: 3.2.0; https://satijalab.org/seurat/) with thresholds set to log_2_ fold change > 0.25 and adjusted *P* value ≤ 0.05. Kyoto Encyclopedia of Genes and Genomes (KEGG) pathway enrichment analysis was performed on up-regulated DEGs with a log₂ fold change > 0.58. Cell–cell interaction analysis was carried out using the CellPhoneDB database (https://github.com/ventolab/cellphonedb-data). Furthermore, the Dr. Tom platform (BGI) was utilized for KEGG pathway analysis, uniform manifold approximation and projection (UMAP) visualization, and violin plot analysis of gene expression from the scRNA-seq data.

### Statistical analysis

All experiments were repeated at least 3 times, and data were presented as mean ± standard deviation (SD). Statistical analysis was performed with GraphPad software (version 8.0; https://www.graphpad.com). The significance of the statistical difference between the 2 groups was analyzed using Student’s *t* test, while 1-way analysis of variance (ANOVA) and 2-way ANOVA were used for analyzing multiple groups. Significance was determined for *P* values less than 0.05.

## Results

### The heterogeneity of neutrophils in BC lung metastatic niches

Immune cells are known to play important roles in cancer metastasis. To characterize the immune landscape within the lung microenvironment during BC lung metastasis, we established lung-tropic metastatic models using 4T1-LM3 and E0771-LM3 BC cells derived through continuous in vivo selection and amplification from their parental lines (Fig. S1A). Through serial selection of lung-tropic subpopulations, we observed the enhanced lung-specific metastatic propensity in mice (Fig. S1B and C). We then established spontaneous metastasis models by implanting 4T1-LM3 or E0771-LM3 cells into the mammary fat pads of BALB/c or C57BL/6 mice, respectively.

Ccl2, Mmp9, and S100a9 were generally considered as biomarkers of premetastatic niche formation [2]. In the 4T1-LM3 model, micrometastatic lesions were identified in the lungs at 3 weeks after cell injection, and larger macrometastases were observed after 4 weeks (Fig. S1D). Notably, at 2 weeks, although no metastatic cells were detected, there was increased expression of Ccl2, Mmp9, and S100a9 in the lungs of tumor-bearing mice compared with normal lung tissues (Fig. S2A). Based on these findings, the second week post-injection of 4T1-LM3 cells was defined as the premetastatic stage, with the third week as the micrometastatic stage, and the fourth week or beyond as the macrometastatic stage. Similarly, in C57BL/6 mice injected with E0771-LM3 cells, weeks 1, 2, and 3 were designated as the pre-, micro-, and macrometastatic stages, respectively (Figs. S1D and S2B).

**Fig. 1.**
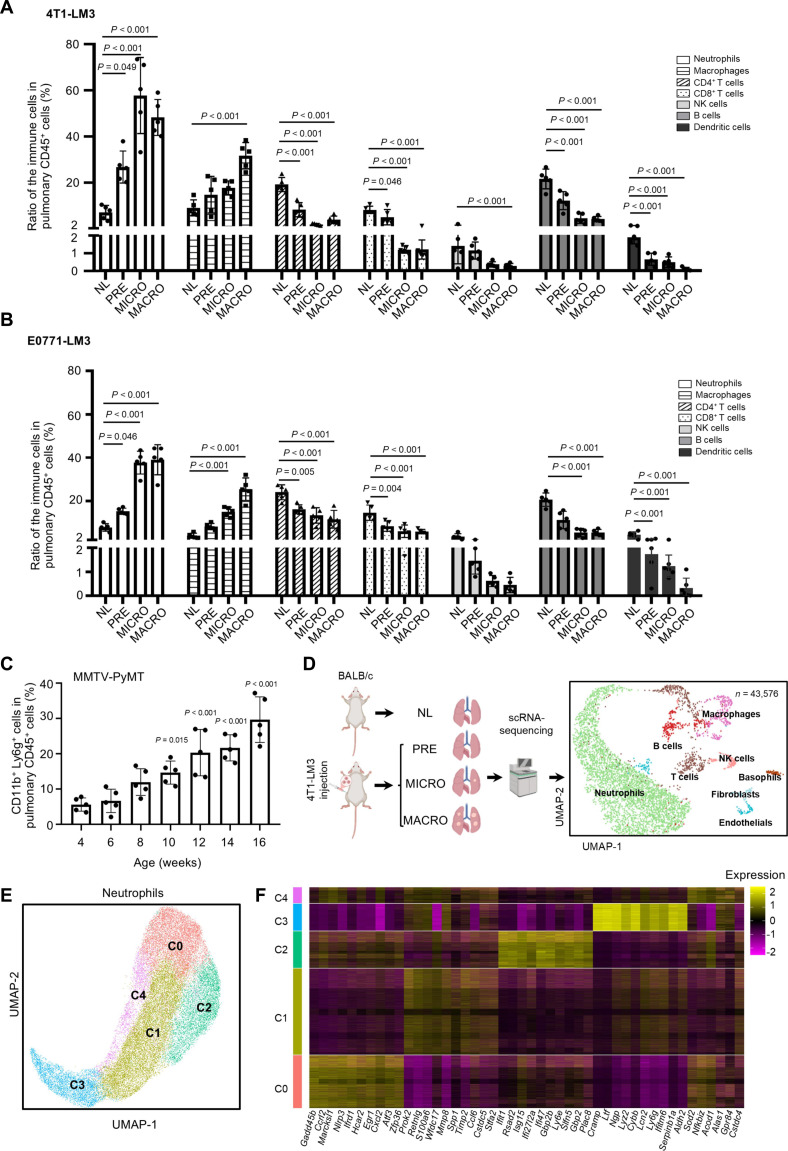
The heterogeneous neutrophils in the lung metastatic niche. (A and B) Flow cytometry analysis of lung immune cell populations in BALB/c mice inoculated with 4T1-LM3 cells [4T1-LM3 (BALB/c) model, *n* = 5, (A)] and C57BL/6 mice inoculated with E0771-LM3 cells [E0771-LM3 (C57BL/6) model, *n* = 5, (B)]. Statistical significance was determined by comparison with immune cell populations in normal lungs. (C) Quantification of pulmonary neutrophils (CD11b^+^Ly6g^+^CD45^+^) in MMTV-PyMT mice at various weeks of age (*n* = 5). Statistical significance was determined by comparison with neutrophils from the lungs of control mice at 4 weeks of age. (D) Schematic diagram illustrating the experimental approach, and UMAP visualization of cell clusters color-coded by cell type with a dot plot (NL: *n* = 2 tissues, 11,175 cells; PRE: *n* = 2 tissues, 10,357 cells; MICRO: *n* = 2 tissues, 12,938 cells; MACRO: *n* = 2 tissues, 9,106 cells; total number of cells: *n* = 43,576). (E) UMAP plot of neutrophils in the normal and metastasis-related lung tissues from the 4T1-LM3 (BALB/c) model, color-coded by associated cluster. (F) Heatmap showing the expression of top 10 marker genes for each neutrophil cluster (yellow, high expression; purple, low expression). The data with error bars are presented as the mean ± SD; statistical significance was determined by 2-way ANOVA (A and B) and 1-way ANOVA test (C). 4T1-LM3, 4T1-lung metastasis 3; ANOVA, analysis of variance; CD4, cluster of differentiation 4; CD8, cluster of differentiation 8; E0771-LM3, E0771-lung metastasis 3; Ly6g, lymphocyte antigen 6 complex locus g; MACRO, macrometastatic lung; MICRO, micrometastatic lung; MMTV-PyMT, mouse mammary tumor virus-polyomavirus middle T antigen; NK, natural killer; NL, normal lung; PRE, premetastatic lung; scRNA-seq, single-cell RNA sequencing; SD, standard deviation; UMAP, uniform manifold approximation and projection.

We next profiled the dynamics of key immune cell composition in the lung metastatic niche. Flow cytometry revealed significant neutrophil accumulation as early as the premetastatic stage, with neutrophils becoming the predominant immune population during micro- and macrometastasis (Fig. 1A and B). This pattern was corroborated in the MMTV-PyMT mammary tumor mouse model (Fig. 1C). To further resolve the immune landscape, we performed scRNA-seq on the lungs across metastatic stages (Fig. 1D). Most immune cell clusters, including basophils, B cells, macrophages, neutrophils, T cells, and NK cells, were identified using their marker genes [17,18] (Fig. S2C). Consistent with previous reports [18,19], neutrophils constituted the most abundant immune subset in the lung-specific metastatic niche, whereas the proportions of T cells, NK cells, and B cells decreased over time (Fig. S2D).

Notably, 5 heterogeneous neutrophil subsets were identified from the integrated dataset, which included lung tissues from normal and different metastatic stages (pre-, micro-, and macrometastatic), and were classified into clusters C0 to C4. Cluster C0 highly expressed cell apoptosis-related genes such as growth arrest and DNA damage-inducible 45 (*Gadd45b*), nucleotide-binding oligomerization domain-like receptor protein 3 (*Nlrp3*), hydroxycarboxylic acid receptor 2 (*Hcar2*), and zfp36 ring finger protein (*Zfp36*) [20–22]. Cluster C1 highly expressed adhesion and migration genes, such as resistin like gamma (*Retnlg*), wap four-disulfide core domain 17 (*Wfdc17*), secreted phosphoprotein 1 (*Spp1*), and c-c motif ligand 6 (*Ccl6*) [23]. Cluster C2 highly expressed interferon-response signature genes, such as interferon-induced protein with tetratricopeptide repeats 1 (*Ifit1*), radical s-adenosyl methionine domain containing 2 (*Rsad2*), and interferon-stimulated gene 15 (*Isg15*) [24]. Cluster C3 highly expressed mature-associated markers, such as *Cramp* and lymphocyte antigen 6 complex locus g (*Ly6g*), whereas cluster C4 expressed mitochondria-related genes, such as superoxide dismutase (*Sod2*), aconitate decarboxylase 1 (*Acod1*), and 5′-aminolevulinate synthase 1 (*Alas1*) [25,26] (Fig. 1E and F). In summary, neutrophils accumulate early in the lung premetastatic niche and emerge as the dominant and heterogeneous immune populations throughout BC lung metastasis, highlighting their central role in the metastatic immune microenvironment.

### Ly6g^low^ neutrophils possess the gene expression feature of myeloid-derived suppressor cells

**Fig. 2.**
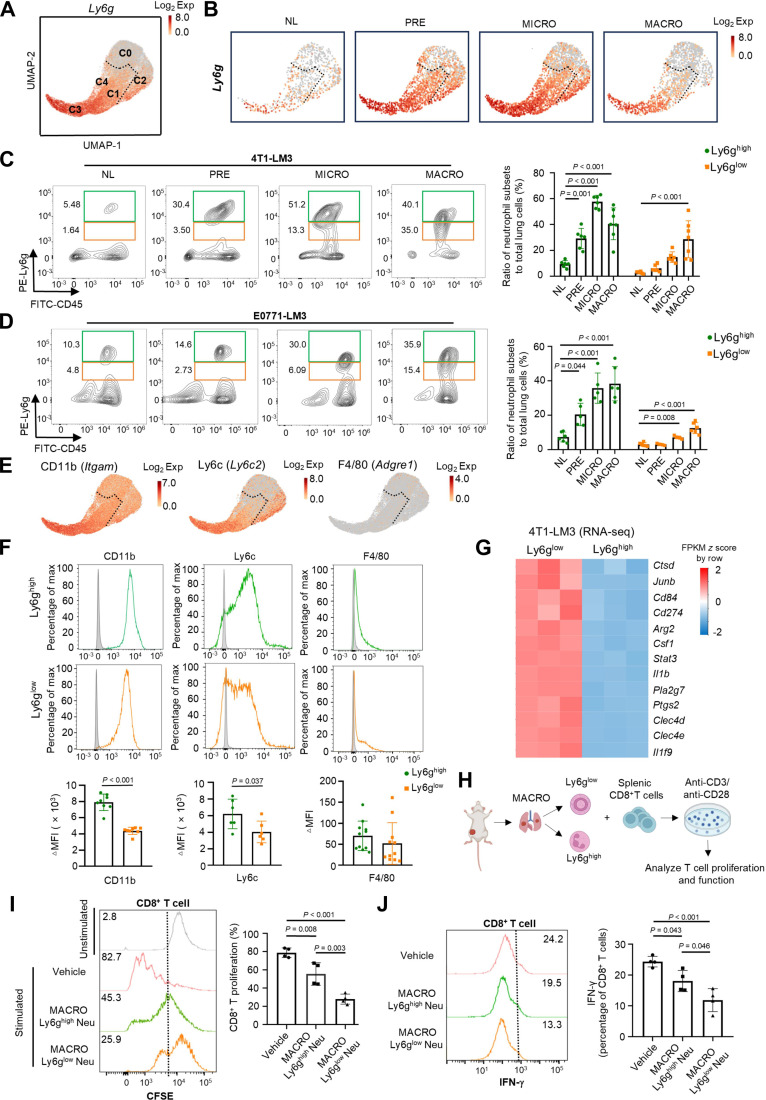
Dynamic changes in Ly6g^high^ and Ly6g^low^ neutrophil subsets in the lung metastatic microenvironment. (A and B) UMAP plots showing *Ly6g* expression of neutrophil subsets derived from integrated data of lung tissue (A) and lung tissues at different stages of BC metastasis (B) [4T1-LM3 (BALB/c) model]. The dashed line separates the Ly6g^low^ (above) and Ly6g^high^ (below) neutrophil subsets. (C and D) Representative fluorescence-activated cell sorting (FACS) plot of Ly6g^high^ and Ly6g^low^ neutrophils in normal lung tissue and lung tumor tissue at different metastatic stages in breast tumor-bearing mice [4T1-LM3 (BALB/c) model: NL (*n* = 6), PRE (*n* = 6), MICRO (*n* = 6), MACRO (*n* = 7), (C); E0771-LM3 (C57BL/6) model: NL (*n* = 6), PRE (*n* = 5), MICRO (*n* = 5), MACRO (*n* = 6), (D)]. The bar graph on the right shows the quantification of the proportions of Ly6g^high^ and Ly6g^low^ neutrophil subsets in lung tissues during BC pulmonary metastasis. Statistical significance was determined by comparison with NL. (E) UMAP plots of neutrophils, color-coded for the expression level of genes encoding CD11b (*Itgam*), Ly6c (*Ly6c2*), and cell surface glycoprotein F4/80 (*Adgre1*). The dashed line separates the Ly6g^low^ (above) and Ly6g^high^ (below) neutrophil subsets. (F) Expression levels of myeloid markers in Ly6g^high^ (top) and Ly6g^low^ (bottom) neutrophils, including CD11b (*n* = 8), Ly6c (*n* = 6), and F4/80 (*n* = 11). (G) Heatmap displaying the expression level of the genes related to immunosuppression based on the RNA-seq data. (H to J) As depicted in the schematic, Ly6g^high^ and Ly6g^low^ neutrophils were separated from the macrometastatic lung tissues [4T1-LM3 (BALB/c) model] and were respectively cocultured with naïve splenic CD8^+^ T cells at a ratio of 1:1 in the presence of plate-bound anti-CD3 antibody and soluble anti-CD28 antibody to facilitate T cell activation (H). T cell proliferation was quantified by flow cytometry [*n* = 4, (I)]; IFN-γ was determined using intracellular staining by flow cytometry [*n* = 4, (J)]. The data with error bars are presented as the mean ± SD; statistical significance was determined by 2-way ANOVA (C and D), Student’s *t* test (F), and 1-way ANOVA test (I and J). 4T1-LM3, 4T1-lung metastasis 3; △MFI, the difference of mean fluorescence intensity; *Adgre1*, adhesion g protein-coupled receptor e1; ANOVA, analysis of variance; *Arg2*, arginase 2; BC, breast cancer; CD3, cluster of differentiation 3; *Cd11b*, integrin subunit α m; CD11b, integrin α-m; *Cd274*, cluster of differentiation cd274; CD28, cluster of differentiation 28; *Cd84*, cluster of differentiation 84; *Clec4d*, c-type lectin domain family 4 member d; *Clec4e*, c-type lectin domain family 4 member e; *Csf1*, colony-stimulating factor 1; *Ctsd*, cathepsin d, E0771-LM3, E0771-lung metastasis 3; Exp, expression; FITC, fluorescein isothiocyanate; IFN-γ, interferon-γ; *Il1b*, interleukin 1β; *Il1f9,* interleukin 1 family member 9; FPKM ,fragments per kilobase million; *Itgam,* integrin subunit α M; *Junb*, junb proto-oncogene; Ly6c, lymphocyte antigen c2; *Ly6c2*, lymphocyte antigen c2; Ly6g, lymphocyte antigen 6 complex locus g; MACRO, macrometastatic lung; MICRO, micrometastatic lung; Neu, neutrophils; NL, normal lung; PE, phycoerythrin; *Pla2g7*, phospholipase a2 group vii; PRE, premetastatic lung; *Ptgs2*, prostaglandin-endoperoxide synthase 2; RNA-seq, RNA sequencing; SD, standard deviation; *Stat3*, signal transducer and activator of transcription 3; UMAP, uniform manifold approximation and projection.

Ly6g, a typical neutrophil marker, is differentially expressed among neutrophil subsets within the lung mTME [27]. Based on scRNA-seq data, we observed higher *Ly6g* expression in clusters C1, C3, and C4 compared to C0 and C2 (Fig. 2A and B and Fig. S3A). Accordingly, we classified clusters C1, C3, and C4 as Ly6g^high^ neutrophils, and clusters C0 and C2 were defined as Ly6g^low^ neutrophils. To investigate the dynamic changes of Ly6g^high^ and Ly6g^low^ subpopulations during metastasis, we performed flow cytometry analysis and found that the proportion of Ly6g^high^ neutrophils significantly increased during both the premetastatic and micrometastatic stages, whereas the accumulation of Ly6g^low^ neutrophils occurred predominantly in the macrometastatic stage (Fig. 2C and D). Phenotypic characterization showed that, unlike the relatively immature Ly6g^low^ neutrophils (Fig. S3B), Ly6g^high^ neutrophils expressed higher levels of c-x-c motif chemokine receptor 2 (Cxcr2), a biomarker of mature neutrophils (Fig. S3C and D). Both Ly6g^high^ and Ly6g^low^ subsets were negative for the macrophage marker F4/80 but highly expressed the myeloid markers Cd11b and Ly6c (Fig. 2E and F). To gain deeper insights into neutrophil biology, bulk RNA-seq of Ly6g^high^ and Ly6g^low^ neutrophils from macrometastatic lesions versus Ly6g^high^ neutrophils from normal lung tissue revealed distinct transcriptional landscapes via PCA (Fig. S3E and F). Notably, Ly6g^low^ neutrophils displayed myeloid-derived suppressor cell (MDSC)-related gene expression signatures, including immunosuppressive genes, such as *Cd84* and *Cd274* [28] (Fig. 2G). These gene expression patterns were also validated by scRNA-seq analysis (Fig. S3G), suggesting an immunosuppressive role for Ly6g^low^ neutrophils.

To verify the potential immunosuppressive role of Ly6g^high^ and Ly6g^low^ neutrophils, we isolated both neutrophil subsets from macrometastatic lungs and cocultured them with CD8^+^ T cells in vitro (Fig. 2H). Ly6g^low^ subsets demonstrated a more robust ability to suppress CD8^+^ T cell proliferation and function than Ly6g^high^ neutrophils (Fig. 2I and J). Together, Ly6g stratification reveals functionally distinct neutrophil subsets in the lung metastatic cascade, wherein Ly6g^low^ neutrophils bearing MDSC-like transcriptional signatures, including immunosuppressive genes, exhibit a superior capacity to inhibit the proliferation and effector functions of CD8^+^ T cells.

### Ly6g^high^ neutrophils form NETs to promote lung metastasis of BC

**Fig. 3.**
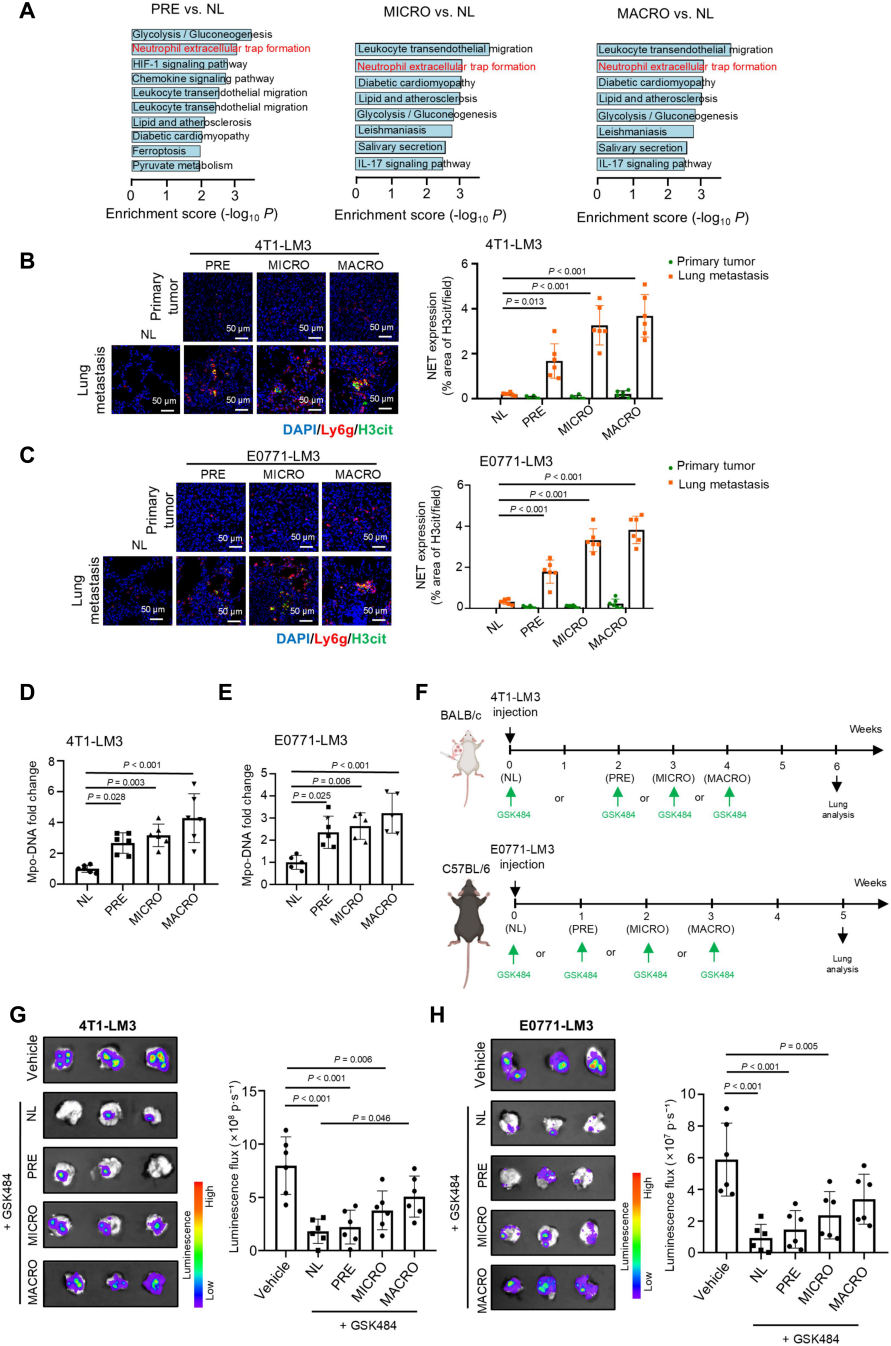
Ly6g^high^ neutrophils promote BC lung metastasis via NETs. (A) KEGG pathway analysis of DEGs (log_2_ fold change > 0.58) in Ly6g^high^ neutrophil subsets from PRE, MICRO, and MACRO phases compared to NL, based on scRNA-seq data [4T1-LM3 (BALB/c) model]. (B and C) Representative immunofluorescence micrographs showing NET formation at the indicated time points of the metastasis stages [4T1-LM3 (BALB/c) model, *n* = 6, (B); E0771-LM3 (C57BL/6) model, *n* = 6, (C)]. NETs were stained with antibodies against Ly6g (red) and H3cit (green), and nuclei were counterstained with DAPI (blue). (D and E) Histogram showing plasma NET levels at different metastatic stages, as evaluated by quantifying plasma MPO–DNA complexes [4T1-LM3 (BALB/c) model, *n* = 6, (D); E0771-LM3 (C57BL/6) model, *n* = 5, (E)]. (F) Schematic overview of the treatment strategy targeting NETs. Briefly, the PADI4 inhibitor GSK484 (20 mg/kg) was administered intraperitoneally on a daily schedule, starting either on the day of tumor cell injection (NL) or at the PRE, MICRO, or MACRO stages. (G and H) Representative bioluminescence images of mice following the therapeutic regimen illustrated in (F). [4T1-LM3 (BALB/c) model, *n* = 6, (G); E0771-LM3 (C57BL/6) model, *n* = 6, (H)]. The bar graph on the right shows the quantification of the luminescence of lung tissues. The data with error bars are presented as the mean ± SD; statistical significance was determined by 2-way ANOVA (B and C) and 1-way ANOVA test (D, E, G, and H). 4T1-LM3, 4T1-lung metastasis 3; ANOVA, analysis of variance; BC, breast cancer; DAPI, 4’,6-diamidino-2-phenylindole; DEGs, differentially expressed genes; E0771-LM3, E0771-lung metastasis 3; H3cit, citrullinated histone H3; KEGG, Kyoto Encyclopedia of Genes and Genomes; Ly6g, lymphocyte antigen 6 complex locus g; MACRO, macrometastatic lung; MICRO, micrometastatic lung; MPO, myeloperoxidase; NETs, neutrophil extracellular traps; NL, normal lung; PADI4, peptidyl arginine deiminase 4; PRE, premetastatic lung; SD, standard deviation.

During the premetastatic stage, Ly6g^high^ neutrophils are significantly recruited to the metastatic lung. However, their functional role remains unclear. To elucidate potential involvement of Ly6g^high^ neutrophils in metastasis, we performed KEGG pathway analysis to determine their biological functions and gene expression profiles across pre-, micro-, and macrometastatic stages compared with normal lung tissue. The results revealed that NET formation potentially played a vital role in Ly6g^high^ neutrophils in the lung metastasis process (Fig. 3A). Consistent with this, we observed marked up-regulation of peptidyl arginine deiminase 4 (*Padi4*), a histone-modifying enzyme and crucial factor for NET formation [29], in metastatic Ly6g^high^ neutrophils. Besides, *Cd177* and olfactomedin 4 (*Olfm4*), which are exclusively present in some neutrophils and are unaffected by the activation or maturity of neutrophils [30–32], were also highly expressed in metastatic Ly6g^high^ neutrophils (Fig. S4A). These findings suggest that metastasis-related Ly6g^high^ neutrophils that accumulate in the lung tissues have a strong ability to form NETs and are potentially involved in BC lung metastasis. We therefore hypothesized that metastatic Ly6g^high^ neutrophils possess a high capacity for NET formation. To test this, we compared the NET release capacity of Ly6g^high^ and Ly6g^low^ neutrophils under the same stimulation conditions. Ly6g^high^ neutrophils exhibited significantly elevated expression of citrullinated histone H3 (H3cit), a specific marker for NETosis [33] (Fig. S4B). Bulk RNA-seq analysis further confirmed enrichment of NET formation pathways in DEGs between macrometastatic and normal Ly6g^high^ neutrophils (Fig. S4C). To confirm the formation of NETs in lung metastases, we conducted immunofluorescence staining to explore Ly6g and H3cit in the metastatic lungs at different stages. It was confirmed that NET formation markedly increased from the premetastatic to the macrometastatic stage, but is hardly observed in both murine and human primary breast tumors (Fig. 3B and C and Fig. S4D). Correspondingly, the levels of NETs in the plasma, measured by MPO–DNA complex as described previously [34], were significantly increased in the peripheral blood of mice from the premetastatic to macrometastatic stages compared to tumor-free mice (Fig. 3D and E). To functionally evaluate the contribution of NETosis to metastasis, we inhibited PADI4 at different metastatic stages (Fig. 3F). Early PADI4 inhibition initiated at tumor cell inoculation or premetastatic stages effectively inhibited NET formation and prevented lung metastasis compared to later interventions during micro- or macrometastatic progression (Fig. 3G and H and Fig. S4E and F). Notably, primary breast tumor growth remained unaffected regardless of treatment onset (Fig. S4G and H). Together, these data demonstrate that early-recruited Ly6g^high^ neutrophils promote BC lung metastasis through NETosis, supporting the targeting of NET formation as a potential strategy for preventing metastatic progression.

### Macrophage-secreted Il-1β endows Ly6g^high^ neutrophils with NETosis ability

**Fig. 4.**
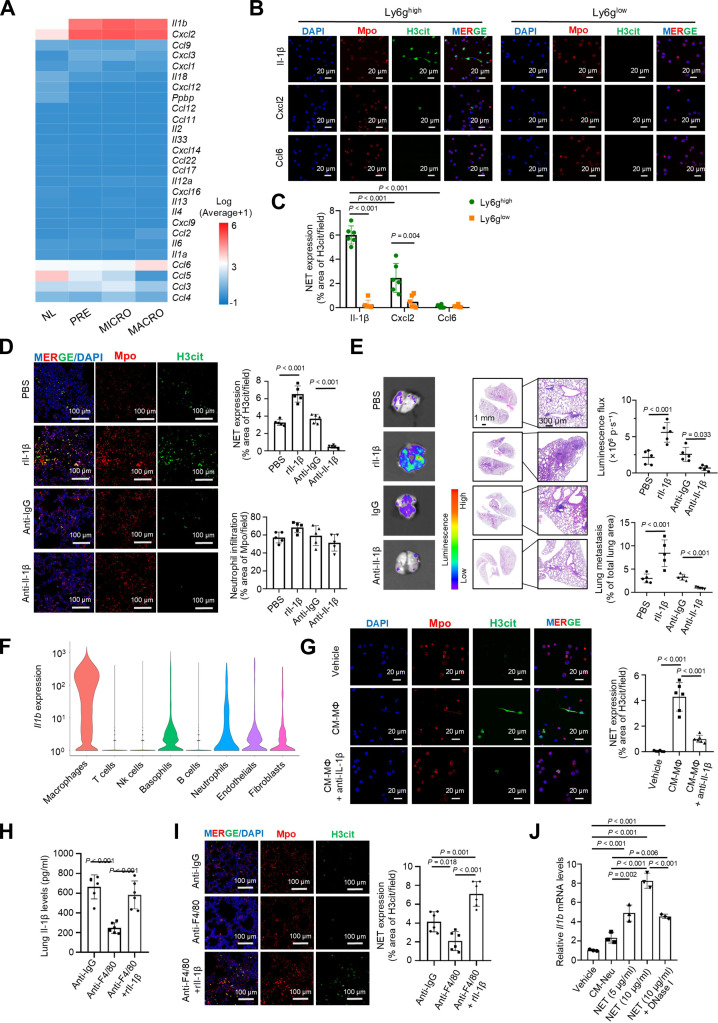
Il-1β induces Ly6g^high^ neutrophil NETosis in the lung metastatic niche. (A) Heatmap of the scRNA-seq data showing the expression of cytokine genes at different time points during lung metastasis. (B and C) Representative immunofluorescence micrographs (B) showing NET formation by FACS-sorted Ly6g^high^ and Ly6g^low^ neutrophils (*n* = 6) after treatment with Il-1β, Cxcl2, and Ccl6 for 6 h in vitro. NETs were stained with antibodies against Mpo (red) and H3cit (green), and nuclei were counterstained with DAPI (blue). The statistical data are presented in (C). (D) Representative immunofluorescence micrographs showing NET formation at the MACRO stages of lung tissue with PBS, rIl-1β, anti-IgG, and anti-Il-1β antibody treatment, respectively [4T1-LM3 (BALB/c) model, *n* = 5]. NETs were stained with antibodies against Mpo (red) and H3cit (green), and nuclei were counterstained with DAPI (blue). The bar graph on the right quantifies NET formation. (E) Representative bioluminescence imaging and hematoxylin and eosin (H&E) staining images at the MACRO lungs from mice treated with PBS, rIl-1β, IgG, or anti-Il-1β antibody [4T1-LM3 (BALB/c) model, *n* = 5]. The bar graph on the right shows the quantitative data of lung metastasis burden. (F) Violin plots showing the expression of *Il1b* in different cell clusters in the lung tissues based on scRNA-seq data from Fig. 1D. (G) Representative immunofluorescence micrographs demonstrate NET formation in sorted Ly6g^high^ neutrophils (*n* = 6). Neutrophils were treated with CM-MΦ or CM-MΦ that had been neutralized with an anti-Il-1β antibody. NETs were stained with antibodies against Mpo (red) and H3cit (green), and nuclei were counterstained with DAPI (blue). (H and I) Mice were treated with anti-IgG control, anti-F4/80 antibody, or anti-F4/80 antibody combined with rIl-1β until the macrometastatic stage [4T1-LM3 (BALB/c) model, *n* = 6]. (H) Il-1β levels in the lungs were detected by ELISA. (I) Representative immunofluorescence images show NET formation. NETs were stained for Mpo (red) and H3cit (green), and nuclei were counterstained with DAPI (blue). The bar graph on the right quantifies NET formation (I). (J) Macrophages were treated with CM-Neu, NETs (5 μg/ml), NETs (10 μg/ml), or NETs (10 μg/ml) combined with deoxyribonuclease (DNase) I (*n* = 3). The expression of *Il1b* was determined by qPCR. The data with error bars are presented as the mean ± SD; statistical significance was determined by 2-way ANOVA (C) and 1-way ANOVA test (D, E, and G to J). 4T1-LM3, 4T1-lung metastasis 3; ANOVA, analysis of variance; *Ccl11*, c-c motif chemokine ligand 11; *Ccl12*, c-c motif chemokine ligand 12; *Ccl17*, c-c motif chemokine ligand 17; *Ccl2*, c-c motif chemokine ligand 2; *Ccl22*, c-c motif chemokine ligand 22; *Ccl3*, c-c motif chemokine ligand 3; *Ccl4*, c-c motif chemokine ligand 4; *Ccl5*, c-c motif chemokine ligand 5; *Ccl6*, c-c motif chemokine ligand 6; CCL6; c-c motif ligand 6; *Ccl9*, c-c motif chemokine ligand 9; CM-MΦ, macrophage-derived conditioned medium; CM-Neu, neutrophil-derived conditioned medium; *Cxcl12*, c-x-c motif chemokine ligand 12; *Cxcl14*, c-x-c motif chemokine ligand 14; *Cxcl16*, c-x-c motif chemokine ligand 16; CXCL2, c-x-c motif chemokine ligand 2; *Cxcl2*, c-x-c motif chemokine ligand 2; *Cxcl3*, c-x-c motif chemokine ligand 3; *Cxcl9*, c-x-c motif chemokine ligand 9; DAPI, 4’,6-diamidino-2-phenylindole; ELISA, enzyme linked immunosorbent assay; FACS, fluorescence-activated cell sorting; H3cit; citrullinated histone H3; *Il12a*, interleukin 12a; *Il13*, interleukin 13; *Il18*, interleukin, 18; *Il1a*, interleukin 1α; *Il1b*, interleukin 1β; Il-1β, interleukin-1β; *Il2*,interleukin 2; *Il33*, interleukin 33; *Il4*, interleukin 4; *Il6*, interleukin 6; Ly6g, lymphocyte antigen 6 complex locus g; MACRO, macrometastatic lung; MICRO, micrometastatic lung; MPO, myeloperoxidase; NETs, neutrophil extracellular trap; NK, natural killer; NL, normal lung; *Ppbp*, pro-platelet basic protein; Neu, neutrophil; PRE, premetastatic lung; qRT-PCR, quantitative real-time polymerase chain reaction; rIl-1β, recombinant interleukin-1β; scRNA-seq: single-cell RNA sequencing; SD, standard deviation.

Cytokine-driven NET formation is a recognized mechanism in cancer metastasis [35,36]. In scRNA-seq analysis, we observed that cytokine-related genes, including *Il1b*, *Cxcl2*, and *Ccl6*, were up-regulated in the lung microenvironment across metastatic stages compared to normal lung tissue, which was further confirmed by ELISA (Fig. 4A and Fig. S5A and B). Among these, Il-1β not only potentiated NET formation more robustly in Ly6g^high^ neutrophils compared to Ly6g^low^ counterparts but also demonstrated superior NET-induction efficacy relative to Cxcl2 and Ccl6 in vitro (Fig. 4B and C). Consistent with these functional observations, IL-1β levels positively correlated with PADI4 expression in human normal lung samples (Fig. S5C) and were associated with NETosis in human BC lung metastases (Fig. S5D). Additionally, administration of rIl-1β to tumor-bearing mice stimulated NET formation and increased lung metastatic burden (Fig. 4D and E and Fig. S5E). These data support the essential role of Il-1β in NET formation in the lung mTME.

To determine the cellular sources of Il-1β in the lung metastatic niche, we first performed gene expression analysis using scRNA-seq data and found that *Il1b* was highly expressed in pulmonary macrophages (Fig. 4F), which was confirmed at the protein level in isolated lung cells (Fig. S5F). To further investigate whether Il-1β secreted by pulmonary macrophages stimulated NET formation, neutrophils were treated with CM from metastatic pulmonary macrophages (CM-MΦ), and NET formation was notably increased, whereas neutralization of Il-1β with an anti-Il-1β antibody inhibited NET formation induced by CM-MΦ in vitro (Fig. 4G). More importantly, the NETs were positively correlated with the macrophages expressing Il-1β in the lung metastatic niche (Fig. S5G). In addition, macrophage depletion in mice reduced Il-1β levels in the metastatic niche, NET formation, and lung metastasis lesions, which were reversed by exogenous supplementation of Il-1β (Fig. 4H and I and Fig. S6A and B). Il-1β has been reported to enhance the NET formation by activating the P38 mitogen-activated protein kinase (MAPK) pathway and triggering ROS production [37]. Consistent with these findings, we found that CM-MΦ induced p38 phosphorylation and ROS production in Ly6g^high^ neutrophils—an effect blocked by anti-Il-1β antibody—while failing to elicit functional responses in Ly6g^low^ neutrophils (Fig. S6C and D). Transcriptomic profiling also revealed higher expression of oxidation-related genes, such as cytochrome b-245 α chain (*Cyba*) and cytochrome b-245 heavy chain (*Cybb*), in Ly6g^high^ neutrophils, compared to Ly6g^low^ neutrophils (Fig. S6E), and cell–cell interaction analysis also displayed a strong communication between macrophages and Ly6g^high^ neutrophils, particularly via macrophage-derived Il-1β signal (Fig. S6F and G). Notably, macrophages treated with NETs showed a significant up-regulation of *Il1b* expression (Fig. 4J), suggesting NET-mediated crosstalk and positive feedback between neutrophils and macrophages. Altogether, our findings demonstrate that Il-1β secretion from macrophages plays a critical role in NETosis by Ly6g^high^ neutrophils in the lung metastatic niche to promote BC lung metastasis.

### Ly6g^high^ neutrophil-derived NETs induced CD8^+^ T cell death via cathelicidin to facilitate BC lung metastasis

**Fig. 5.**
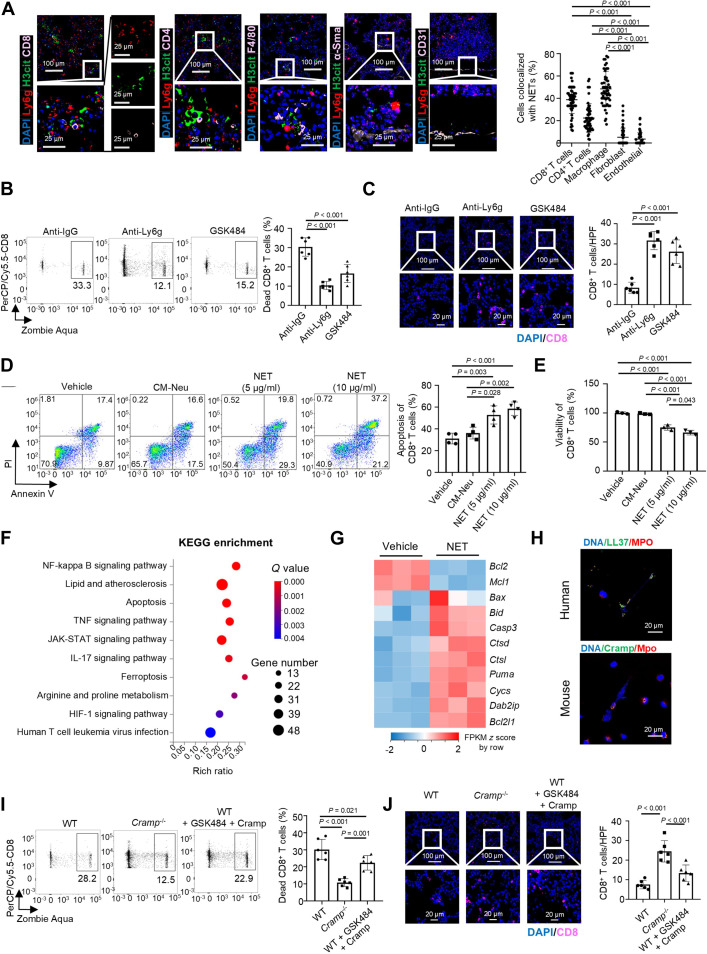
NETs foster BC lung metastasis by inducing the apoptosis of CD8^+^ T cells through cathelicidin. (A) Representative immunofluorescence images showing the colocalization of NETs with CD8^+^ T cells (CD8), CD4^+^ T cells (CD4), macrophages (F4/80), fibroblasts (α-Sma), or endothelial cells (CD31) at the premetastatic stage [4T1-LM3 (BALB/c) model, *n* = 5]. The colocalization was assessed by analyzing 10 fields per mouse. The bar graph on the right quantifies the extent of colocalization of NETs with each cell type in the lung tissue. (B) 4T1-LM3 (BALB/c) mouse model (*n* = 6) was treated with anti-IgG antibody (control), anti-Ly6g antibody, or PADI4 inhibitor (GSK484). CD8^+^ T cells were then isolated from the macrometastatic lung of these mice and incubated with the cell death dye Zombie Aqua. The proportion of dead CD8^+^ T cells was analyzed by FACS. The bar graph quantifies the proportion of dead CD8^+^ T cells in each treatment group. (C) Representative immunofluorescence staining images showing CD8^+^ T cells in metastatic lungs from mice in the indicated treatment. Corresponding quantifications are shown on the right (*n* = 6). (D and E) CD8^+^ T cells were treated with vehicle (cell-free culture medium), CM-Neu, NETs (5 μg/ml), or NETs (10 μg/ml). The apoptosis [*n* = 4, (D)] and viability of CD8^+^ T cells [*n* = 3, (E)] were quantified. (F) CD8^+^ T cells were isolated from the spleens of BALB/c mice and treated with or without NETs (10 μg/ml) for 48 h prior to RNA-seq analysis. KEGG pathway analysis was subsequently performed on the differentially expressed genes (DEGs) with an absolute log_2_ fold change > 0.58. (G) Heatmap showing the expression of apoptosis-related DEGs identified in CD8^+^ T cells following treatment with or without NETs (*n* = 3). (H) Representative immunofluorescence micrographs showing NET formation in mouse and human neutrophils after stimulation with PMA (200 nM). LL37(green) and Cramp (green) are localized on the DNA scaffold of NETs. (I and J) CD8^+^ T cells were isolated at the macrometastatic stage from 3 groups of mice, including WT mice, *Cramp*-deficient mice, and WT mice injected with E0771-LM3 cells and treated with the PADI4 inhibitor (GSK484) and Cramp. (I) CD8^+^ T cells were incubated with cell death dye Zombie Aqua, and the proportion of dead cells was assessed by FACS. (J) Representative immunofluorescence micrographs showing CD8^+^ T cells in macrometastatic lung tissues of each group. The data with error bars are presented as the mean ± SD; statistical significance was determined by one-way ANOVA test (A to E, I, and J). 4T1-LM3, 4T1-lung metastasis 3; ANOVA, analysis of variance; *Bax*, bcl2-associated x protein; *Bcl2*, B cell lymphoma 2; *Bcl2l1*, bcl2-like 1; *Bid*, bh3 interacting-domain death agonist; *Casp3*, caspase 3; CD8, cluster of differentiation 8; CM-Neu, neutrophil-derived conditioned medium; Cramp, cathelicidin antimicrobial peptide; *Ctsd*, cathepsin d; *Ctsl*, cathepsin l; *Cycs*, cytochrome c; *Dab2ip*, dab2 interacting protein; DAPI, 4',6-diamidino-2-phenylindole; E0771-LM3, E0771-lung metastasis 3; FACS, fluorescence-activated cell sorting; FPKM, fragments per kilobase million; H3cit; citrullinated histone H3; HPF, high power field; KEGG, Kyoto Encyclopedia of Genes and Genomes; Ly6g, lymphocyte antigen 6 complex locus g; *Mcl1*, myeloid cell leukemia 1; MPO, myeloperoxidase; PADI4, peptidyl arginine deiminase 4; PerCP/Cy5.5, peridinin chlorophyll protein-cyanine5.5; PI, propidium iodide; PMA, phorbol 12-myristate 13-acetate; *Puma*, p53 up-regulated modulator of apoptosis; RNA-seq, RNA sequencing; SD, standard deviation; α-Sma, α-smooth muscle actin.

Previous studies have focused on bidirectional communication between cancer cells and NETs at the primary tumor site, which fuels tumor cell invasion and metastasis [38,39]. We observed NET assembly in premetastatic lungs prior to DTC colonization (Fig. 3B and C). Therefore, we further investigated whether NETs could act on nontumor cells to affect the lung microenvironment and contribute to lung metastasis in BC. To this end, we explored the spatial relationship between NETs and major cell subsets in the metastatic niche. We found that NETs had close colocalization with CD8^+^ T cells, CD4^+^ T cells, and macrophages, rather than fibroblasts and endothelial cells (Fig. 5A). Interestingly, either the depletion of neutrophils with anti-Ly6g antibody or the inhibition of NET formation with PADI4 inhibitor reduced the death of pulmonary CD8^+^ T cells and increased infiltration of CD8^+^ T cells (Fig. 5B and C). However, depletion of neutrophils or inhibition of NET formation did not affect the death of CD4^+^ T cells and macrophages in the metastatic lung (Fig. S7A and B). In vitro coculture of NETs with CD8^+^ T cells, CD4^+^ T cells, macrophages, or tumor cells revealed that NETs only impaired the viability of CD8^+^ T cells but not the other cells (Fig. 5D and E and Fig. S7C to E), indicating that NET-mediated CD8^+^ T cell death may contribute to Ly6g^high^ neutrophil-driven BC lung metastasis.

To elucidate the mechanism underlying the NET’s effect on CD8^+^ T cell survival, RNA-seq was performed to examine the gene expression changes in CD8^+^ T cells cultured with or without NETs. Transcriptomic analysis revealed profound gene expression changes (Fig. S7F). KEGG pathway analysis revealed that the apoptosis signaling pathway was enriched in CD8^+^ T cells following NET treatment (Fig. 5F). The genes related to proapoptosis, including bcl-2-associated x protein (*Bax*), bcl-2 homology 3 interacting domain death agonist (*Bid*), caspase 3 (*Casp3*), p53 up-regulated modulator of apoptosis (*Puma*)*,* and cytochrome c (*Cycs*), were significantly up-regulated, whereas the genes associated with anti-apoptosis, such as B cell lymphoma-2 (*Bcl2*) and myeloid cell leukemia-1 (*Mcl1*), were markedly decreased by NET treatment (Fig. 5G). Spatial correlation analysis further confirmed a strong positive correlation between NET density and CD8^+^ T cell apoptosis in metastatic lung tissues, with most apoptotic CD8^+^ T cells located within or near NET-rich regions (Fig. S7G to I). These data suggest that NETs potentially foster BC lung metastasis by inducing CD8^+^ T cell death and reducing the infiltration of CD8^+^ T cells in the lung mTME.

NETs are assembled from DNA fragments coated with histones and toxic granule proteins, including MPO, H3cit, NE, MMP9, and cathelicidin [LL37 (the human form) or Cramp (the murine homolog)] [40,41]. As LL37 is cytotoxic to epithelial cells and B lymphocytes [42], we reasoned that NET-derived LL37 or Cramp plays a role in CD8^+^ T cell apoptosis. Initial validation established the localization of LL37 and Cramp on DNA scaffolds within NETs (Fig. 5H). Treatment with recombinant Cramp or LL37 significantly induced apoptosis in mouse and human CD8^+^ T cells, respectively (Fig. S8A and B). To further determine the role of cathelicidin in the NET-induced apoptosis of CD8^+^ T cells, the mouse-derived CD8^+^ T cells were treated with NETs isolated from the wild-type mice (WT NETs), the *Cramp-*deficient mice (*Cramp*^−/−^ NETs), or WT NETs neutralized with antibodies against Ne or Mpo. It was found that loss of Cramp led to reduced CD8^+^ T cell apoptosis in comparison with that treated with WT NETs or neutralized NETs (Fig. S8C), suggesting Cramp to be necessary for NET-induced CD8^+^ T cell death. Notably, the loss of Cramp did not impair NET formation in the pulmonary microenvironment of *Cramp*^−/−^ mice, but significantly reduced apoptotic CD8^+^ T cells colocalized with NETs (Fig. S8D and E). Compared with untreated *Cramp*^−/−^ mice, WT mice receiving combinatorial PADI4 inhibitor and Cramp therapy exhibited augmented CD8^+^ T cell death, diminished CD8^+^ T cell infiltration in lung tissue, and exacerbated lung metastasis (Fig. 5I and J and Fig. S8F and G). However, primary tumor volume remained unaltered by this therapeutic regimen (Fig. S8H). Together, these results indicate that cathelicidin is a key cytotoxic component of NETs that selectively induces CD8^+^ T cell apoptosis, thereby conducive to lung metastasis of BC.

### Cathelicidin binding to Ant1 induces mitochondrial permeability transition pore opening and promotes CD8^+^ T cell apoptosis

**Fig. 6.**
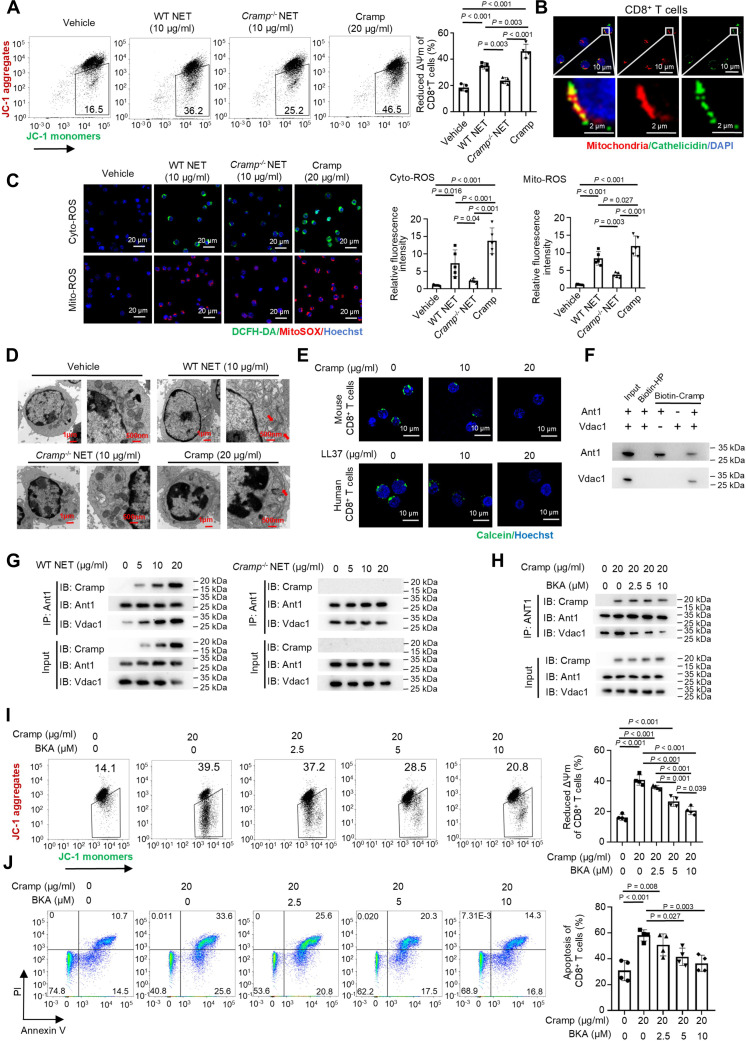
Cathelicidin binding to ANT1 induces mPTP opening and promotes apoptosis in CD8^+^ T cell. (A) CD8^+^ T cells isolated from WT mice were treated with NETs derived from WT or *Cramp*^−/−^ mice, or with Cramp, and then stained with JC-1. ΔΨm was determined by flow cytometry (*n* = 4). The bar graph on the right shows the decreased ΔΨm of CD8^+^ T cells. (B) Representative immunofluorescence images showing colocalization of Cramp (green) with mitochondria (red) in CD8^+^ T cells by confocal laser microscopy. (C and D) CD8^+^ T cells were treated with vehicle (cell-free culture medium), NETs derived from WT or *Cramp*^−/−^ mice, or Cramp. Cytoplasmic and mitochondrial ROS were measured using DCFH-DA and MitoSOX red probes [*n* = 5, (C)]. Representative transmission electron microscopic images of mitochondrial ultrastructure in CD8^+^ T cells under the indicated treatments (D). Red arrows point to mitochondria exhibiting swelling, disorganized cristae, and loss of membrane integrity. (E) Mouse or human CD8^+^ T cells were treated with Cramp or LL37. The mPTP opening of mitochondria was measured by calcein loading/CoCl_2_ quenching. (F) Protein–protein interactions were assessed in vitro using a pull-down assay. Recombinant proteins Ant1 (or Vdac1) and biotin-labeled Cramp (or a cecropin-like antibacterial peptide from *Helicobacter pylori*) were used for this purpose. (G) Co-IP was used to check the interaction between mitochondrial protein Ant1 and Vdac1 in mouse CD8^+^ T cells under the treatment with different concentrations of NETs derived from WT and *Cramp*^−*/*−^ mice (*n* = 3). (H and I) Mouse CD8^+^ T cells were treated with Cramp or Cramp combined with different concentrations of BKA (*n* = 4). (H) The interaction between mitochondrial protein Ant1 and Vdac1 in CD8^+^ T cells was assessed by Co-IP. (I) The ΔΨm of mitochondria in CD8^+^ T cells was assessed by flow cytometry after staining with JC-1. (J) CD8^+^ T cells were treated as in (I), and CD8^+^ T cell apoptosis was quantified by flow cytometry (*n* = 4). The bar graph on the right shows the apoptosis of CD8^+^ T cells. The data with error bars are presented as the mean ± SD; statistical significance was determined by one-way ANOVA test (A, C, I, and J). ANOVA, analysis of variance; Ant1, adenine nucleotide translocator 1; BKA, bongkrekic acid; CD8, cluster of differentiation 8; Co-IP, coimmunoprecipitation; Cramp, cathelicidin antimicrobial peptide; Cyto, cytoplasm; DAPI, 4’,6-diamidino-2-phenylindole; DCFH-DA, 2′,7′-dichlorodihydrofluorescein diacetate; Hp, *Helicobacter pylori*; kDa, kilodalton; Mito, mitochondrial; mPTP, mitochondrial permeability transition pore; NETs, neutrophil extracellular traps; PI, propidium iodide; ROS, reactive oxygen species; Vdac1, voltage dependent anion channel 1; WT, wild type; SD, standard deviation; ΔΨm, mitochondrial membrane potential.

Next, we investigated the mechanism by which NET-derived cathelicidin causes CD8^+^ T cell apoptosis. First, we tested the effects of NETs and Cramp on caspase-8 activity. NETs from WT or Cramp-knockout mice, or mice that received Cramp treatment did not alter the enzymatic activation of caspase-8 (Fig. S9A), suggesting that the extrinsic apoptotic pathway was not involved. However, upon treatment of CD8^+^ T cells, both WT NETs and Cramp significantly reduced ΔΨm, whereas *Cramp*^−/−^ NETs did not (Fig. 6A). A similar effect was noted in human CD8^+^ T cells exposed to LL37 or NETs derived from human neutrophils (Fig. S9B), suggesting a mitochondria-dependent apoptotic mechanism. A previous study revealed that cathelicidin can permeabilize microbial membranes to induce mitochondrial depolarization in B lymphocytes [42], indicating that it plays a direct role in mitochondrial function. Immunofluorescence staining showed that Cramp or LL37 colocalized with mitochondria in mouse or human CD8^+^ T cells (Fig. 6B and Fig. S9C). Mitochondrial dysfunction and the decrease in ΔΨm are known to impair the uncoupling of the electron transport chain and lead to an increase in the production of ROS [43]. As expected, in CD8^+^ T cells treated with WT NETs and either Cramp or LL37, the production of cyto- and mito-ROS was remarkably increased; mitochondria were swollen, mitochondrial cristae were disordered, and mitochondrial membrane integrity was lost (Fig. 6C and D and Fig. S9D). These data suggest that cathelicidin in NETs affects mitochondrial function and induces CD8^+^ T cell apoptosis.

ΔΨm regulates the opening of the mitochondrial permeability transition pore (mPTP), a central event in the process of mitochondria-mediated apoptosis [44]. Therefore, we studied mPTP opening in CD8^+^ T cells and found that cathelicidin treatment effectively decreased the fluorescence intensity, which represents the degree of mPTP opening, indicating that cathelicidin caused mPTP opening in CD8^+^ T cells (Fig. 6E). The mPTP in mice comprises 3 core components: Vdac1/2 in the outer mitochondrial membrane, Ant1/2 in the inner membrane, and cyclophilin d (Cypd) in the matrix, all of which are involved in maintaining ΔΨm and permeability [45]. Next, we knocked down *Vdac1/2*, *Ant1/2*, and Cypd (*Ppid*) in CD8^+^ T cells (Fig. S9E). Loss of Vdac1 and Ant1 reversed the Cramp-induced ΔΨm decrease (Fig. S9F). In the in vitro protein interaction assay, Cramp was found to directly bind to Ant1 but not Vdac1, while Cramp could only restore Vdac1 function in the presence of ANT1 (Fig. 6F). The function of ANT1 in ΔΨm maintenance and mPTP opening is closely dependent on its conformational status: the matrix state, which transports adenosine triphosphate (ATP) out of the matrix to release adenosine diphosphate (ADP) and decreases the binding between ANT1 and VDAC1, and the cytosolic state, which transports ADP into the matrix to release ATP and increases the interaction between ANT1 and VDAC1 [46,47]. To investigate whether Cramp-mediated mPTP opening in CD8^+^ T cells is dependent on the conformation of Ant1, CD8^+^ T cells were treated with WT NETs or *Cramp*^−*/*−^ NETs at graded concentrations. *Cramp*^−*/*−^ NETs failed to alter Ant1–Vdac1 binding, whereas WT NETs progressively enhanced it with increasing concentrations (Fig. 6G). It is known that carboxyatractyloside anchoring of ANT1 in the cytosolic state and BKA treatment in the matrix state control the binding of ANT1 and VDAC1, thereby affecting mPTP opening [47]. Therefore, we administered CD8^+^ T cells with BKA and found that BKA administration inhibited the binding of Ant1 and Vdac1, which reversed the ΔΨm decrease and reduced the cathelicidin-induced CD8^+^ T cell apoptosis (Fig. 6H to J and Fig. S9G and H). Mitochondrial stress and oxidative phosphorylation levels were significantly higher in CD8^+^ T cells treated with WT NETs or Cramp than in control cells or those treated with BKA (Fig. S9I). Critically, compared with untreated controls, BKA treatment reduced CD8^+^ T cell apoptosis while enhancing CD8^+^ T cell infiltration within lung metastatic niches (Fig. S9J and K). Together, these data reveal that NET-derived cathelicidin promotes the formation of the Ant1/Vdac1 complex and thus the mPTP opening, leading to CD8^+^ T cell apoptosis.

### Prognostic significance of NETs and LL37 in patients with BC

**Fig. 7.**
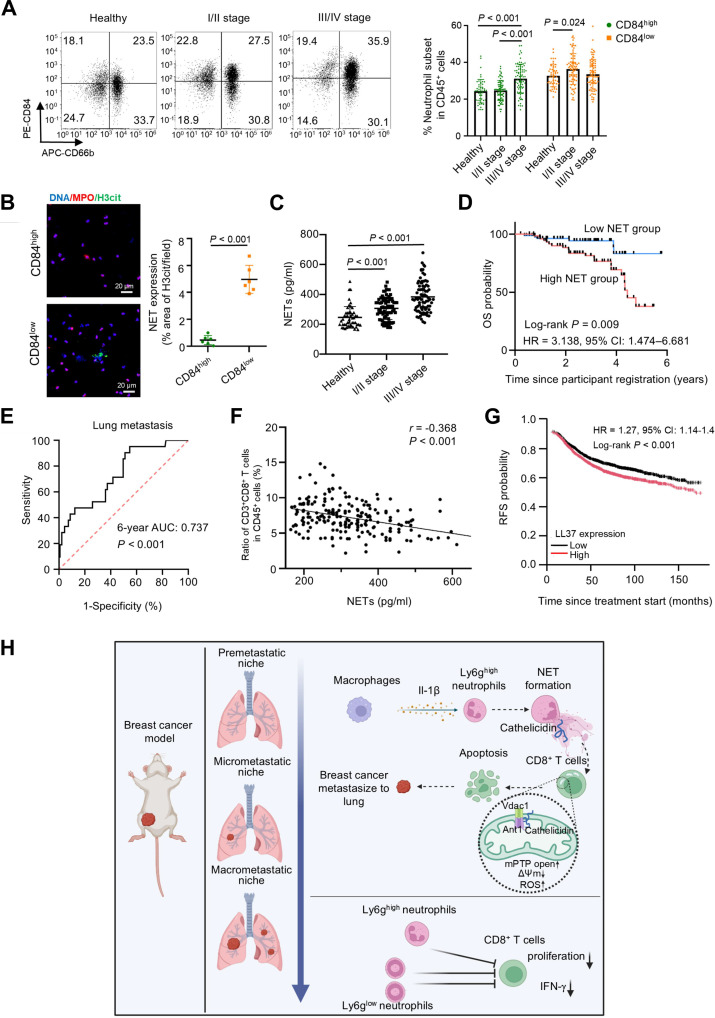
Prognostic significance of NETs in human BC. (A) Representative FACS plot showing the ratio of human CD84^high^ and CD84^low^ neutrophils in healthy individuals (*n* = 50) and patients with BC at different stages [stages I/II (*n* = 80), stages III/IV (*n* = 80)]. To define the CD84^high^ and CD84^low^ subsets in humans, we first established the positive gating threshold using FMO controls. Subsequently, the boundary between “high” and “low” subsets was determined based on a clear inflection point observed in the fluorescence intensity histogram. Statistical significance was determined by comparing with the healthy group. The bar graph on the right quantifies the ratio of human CD84^high^ and CD84^low^ neutrophils. (B) Representative immunofluorescence micrographs showing NET formation of CD84^high^ and CD84^low^ neutrophils, which were sorted by FACS after treatment with PMA for 2 h (*n* = 6). NETs were stained with antibodies against MPO (red) and H3cit (green), and nuclei were counterstained with DAPI (blue). The bar graph on the right quantifies the formation of NETs. (C) Plasma NET levels in healthy individuals (*n* = 50) and BC patients at different stages [stages I/II (*n* = 80), stages III/IV (*n* = 80)]. (D) Kaplan–Meier survival curves showing the overall survival (OS) of BC patients with low (NETs < 344.91 pg/ml; *n* = 83) or high (NETs ≥ 344.91 pg/ml; *n* = 77) concentrations of plasma NETs. BC patients were stratified into high and low NET groups using the mean plasma NET level of the entire cohort as the cutoff. (E) Receiver operator characteristic (ROC) curve analysis of plasma NET levels for predicting BC patients’ lung metastases (*n* = 160). The area under the curve (AUC) value reflects the model’s power to distinguish between BC patients with and without lung metastasis within 6 years after diagnosis. Higher AUC values (approaching 1) denote superior differentiation accuracy at this time point. (F) Correlation between plasma NET levels and CD8^+^ T cell proportion in healthy individuals and patients with BC (*n* = 210). (G) Kaplan–Meier analysis showing the recurrence-free survival of BC patients with high or low levels of LL37 (*n* = 4,929). Data were obtained from the Kaplan–Meier plotter database, which does not provide detailed numerical thresholds for LL37 level classification. (H) Mechanism scheme of Ly6g^high^ and Ly6g^low^ neutrophils in promoting pulmonary metastasis of BC. Briefly, Ly6g^high^ neutrophils accumulated in the premetastatic stage and induced CD8^+^ T cell apoptosis through NETosis. The NET-derived cathelicidin directly bound with Ant1, an mPTP protein in CD8^+^ T cells, leading to conformational changes in the Ant1 and subsequent Ant1–Vdac1 complex formation, which resulted in mPTP opening, loss of ΔΨm, and uncoupling of mitochondrial electron transport chain in CD8^+^ T cells. Ly6g^low^ neutrophils bearing MDSC-like transcriptional signatures exhibit a superior capacity to inhibit the proliferation and effector functions of CD8^+^ T cells. The data with error bars are presented as the mean ± SD; statistical significance was determined by 2-way ANOVA (A), Student’s *t* test (B), 1-way ANOVA test (C), and 2-sided log-rank test (D and G). 4T1-LM3, 4T1-lung metastasis 3; ANOVA, analysis of variance; APC, allophycocyanin; BC, breast cancer; CD8, cluster of differentiation 8; CD84, cluster of differentiation 84; CI, confidence interval; DAPI, 4',6-diamidino-2-phenylindole; E0771-LM3, E0771-lung metastasis 3; FACS, fluorescence-activated cell sorting; FMO, fluorescence-minus-one; H3cit, citrullinated histone H3; HR, hazard ratio; Interferon-γ, IFN-γ; Il-1β, interleukin-1β; Ly6g, lymphocyte antigen 6 complex locus g; MDSC, myeloid-derived suppressor cell; MPO, myeloperoxidase; mPTP, mitochondrial permeability transition pore; NETs, neutrophil extracellular traps; PADI4, peptidyl arginine deiminase 4; PE, phycoerythrin; PMA, phorbol 12-myristate 13-acetate; RFS, recurrence-free survival; ROS, reactive oxygen species; Vdac1, voltage-dependent anion channel 1; SD, standard deviation; ΔΨm, mitochondrial membrane potential.

Our findings indicated that Ly6g^high^ and Ly6g^low^ neutrophils may be used as indicators for the prognosis of patients with BC. CD84 has been considered a surface marker of MDSCs in patients with BC [28]. Interestingly, our study revealed that Ly6g^low^ neutrophils exhibited high CD84 expression, whereas Ly6g^high^ neutrophils displayed low CD84 expression. These findings suggest that CD84 may serve as a marker for distinguishing neutrophil subsets in human samples. The numbers of CD84^high^ neutrophils (CD66b^+^CD84^high^) were increased in the peripheral blood of stage III/IV BC patients compared to healthy volunteers (Fig. 7A). More importantly, CD84^high^ neutrophils highly expressed immunosuppressive genes (Fig. S10A). In addition, CD84^low^ neutrophils (CD66b^+^CD84^low^) in BC patients exhibited a stronger NET-forming ability than CD84^high^ neutrophils (Fig. 7B). Compared with healthy volunteers, the plasma NET levels of BC patients were significantly higher, especially in patients with advanced clinical stage (Fig. 7C). Notably, higher levels of NETs in patients with BC were not only an indicator of poor prognosis but also an independent variable associated with subsequent lung metastasis (Fig. 7D and E). We further revealed a significant negative correlation between plasma NET levels and proportion of CD8^+^ T cells in BC patients (Fig. 7F). High levels of LL37, the key NET-derived factor that causes CD8^+^ T cell apoptosis, were also associated with poor prognosis (Fig. 7G).

Immunotherapy has been demonstrated to prolong the survival of patients with various solid tumors, including metastatic BC [48]. Currently, the common immunotherapeutic approach involves immune checkpoint inhibitors (ICIs), which enhance the cytotoxicity and proliferative capacity of tumor-infiltrating lymphocytes by blocking immunosuppressive receptors. While some BC patients benefit from ICI monotherapy, such as PD-1 targeted therapy, its efficacy remains moderate [49]. To further evaluate the therapeutic potential of NET inhibition in combination with immunotherapy for BC lung metastasis, we treated tumor-bearing mice with a PADI4 inhibitor and an anti-PD-1 monoclonal antibody (Fig. S10B). This combination regimen significantly suppressed lung metastasis compared to anti-PD-1 monotherapy (Fig. S10C and D), further highlighting the potential of targeting NETs as a therapeutic strategy against BC lung metastasis. Taken together, our work demonstrated an essential role of Ly6g^high^ neutrophils in immunosuppressive TME via NET formation to foster BC lung metastasis.

## Discussion

Distant organ metastases from solid tumors cause over 90% of cancer-related deaths [50]. In metastatic tissues, the immunosuppressive microenvironment participates in the formation of metastatic foci by disseminated cancer cells [51]. However, immunosurveillance loss and immune escape, regulated by complex components and their interactions in the mTME, remain poorly understood. By unbiasedly analyzing immune cells with scRNA-seq, we found that pulmonary neutrophils in the mTME can be classified as Ly6g^high^ and Ly6g^low^ subsets. Ly6g^high^ neutrophils accumulated in the premetastatic microenvironment induced CD8^+^ T cell apoptosis through NETosis. NET-derived cathelicidin bound to Ant1 in CD8^+^ T cells, causing conformational changes and forming an Ant1–Vdac1 complex. This opening of mPTP disrupted mitochondrial function and led to CD8^+^ T cell apoptosis. Our findings establish a role for Ly6g^high^ neutrophil in regulating CD8^+^ T cells during BC lung metastasis (Fig. 7H).

Neutrophils exhibit high plasticity and can polarize into distinct subtypes in the TME, previously classified as antitumor N1-type and protumor N2-type [52]. Advances in scRNA-seq have allowed for closer examination of the neutrophil transcriptome in cancers and their interaction with other cells. Several studies have characterized neutrophil populations in the TME of various cancers (e.g., non-small cell lung cancer and pancreatic ductal adenocarcinoma) and their murine models, focusing on tissue- and tumor-specific signatures [53,54]. To date, few studies have thoroughly investigated the heterogeneity of neutrophil populations in the metastatic microenvironment. In a study of human colorectal cancer liver metastasis, tumor-associated neutrophils showed similar signatures to those at the primary site, enriching for markers of neutrophil motility, chemotaxis, and IFN signaling. However, these populations are heterogeneous, with distinct transcriptomic changes linked to T cell suppression [55]. In a pancreatic cancer liver metastasis model, a neutrophil subset (S100a12^+^ neutrophil) was found to be enriched at the invasive front of the metastatic lesions and displayed strong prometastatic properties [56], suggesting that neutrophils undergo further “adaptive” evolution during the metastatic process. By studying the dynamic evolution of immune cell compositions in the BC metastatic lung tissue from the premetastatic niche to the macrometastatic stages in mouse models, we identified 5 neutrophil subsets with distinct transcriptional profiles. Ly6g^low^ neutrophils, which highly express MDSC-related signatures, expanded during the macrometastatic stage. In contrast, Ly6g^high^ neutrophils accumulated in the premetastatic niche and exhibited stronger expression of NET-related genes, suggesting their role in reshaping the mTME. These neutrophil populations are “armed” at metastatic sites and play a key role in shaping organ-specific metastatic microenvironments.

Immunosuppressive TME is a key contributor for DTCs to establish metastasis foci in distant organs. CD8^+^ T cells play a crucial role in mediating immune surveillance; the suppression of their function results in tumor development and increased cancer metastasis [57]. Studies have demonstrated that MDSCs, TAMs, and regulatory T cells can inhibit CD8^+^ T cells in the TME, which contribute to the growth and progression of various primary tumors [58]. Recently, studies have shown cross-regulation of neutrophils and T cells in cancer. Il-17-producing γδ T cells were found to induce expansion and polarization of neutrophils in BC, resulting in suppression of CD8^+^ T cells to boost multi-organ metastasis during the early steps of the metastatic cascade [59]. The up-regulated membranous Nectin2 in tumor-associated neutrophils governs CD8^+^ T cell exhaustion in promoting pancreatic cancer progression [60]. The DNA of NETs binds to the transmembrane and coiled-coil domain 6 on CD8^+^ T cells, inhibiting T cell receptor and nuclear factor κB signaling, thereby impairing antitumor immunity in hepatocellular carcinoma [61]. These works demonstrate that neutrophils directly or indirectly impair CD8^+^ T cells’ function. In the current work, we interestingly found that a large number of neutrophils accumulate in the lung premetastatic niche in BC metastasis mouse models, which has been similarly uncovered by X. Yang in colorectal cancer [62] and by J. Sceneay in mammary and melanoma metastasis models [63]. The predominance of immature neutrophils was also observed in the bone metastasis microenvironment [64], supporting a critical role for neutrophils in organ-preferential metastasis. However, how different subsets of neutrophils promote immunosuppressive mTME remains incompletely understood. In this study, we uncovered that macrophage-derived Il-1β drives pulmonary Ly6g^high^ neutrophils’ NETosis; the cathelicidin (LL37 in humans and Cramp in mice) in NETs has a direct role in inducing CD8^+^ T cell apoptosis in shaping the mTME to promote BC lung metastasis. Blocking the CD8^+^ T cell response to NETs or inhibiting Cramp activity by BKA notably decreased the death of CD8^+^ T cells and increased the infiltration of CD8^+^ T cells in the lung mTME, contributing to less metastasis in tumor-burdened mice. These findings deepen our understanding of the role of Ly6g^high^ neutrophils in promoting CD8^+^ T cell apoptosis in shaping the mTME within BC lung metastatic foci. However, whether other potential components of NETs are involved in CD8^+^ T cell apoptosis remains to be elucidated.

Our studies demonstrate that combining NET inhibition with anti-PD-1 therapy significantly reduces lung metastases in BC models. We also observed progressive NET accumulation in the lung TME during metastasis. Our work suggests that combining neutrophil/NETosis-targeted therapy with ICIs may offer a promising strategy for BC patients with lung metastases. Ly6g^high^ neutrophils and elevated serum NETs could serve as prognostic markers for lung-preferential metastasis. Our findings warrant further research to clarify the regulation of neutrophil subsets in the lung TME and to develop strategies for site-specific NET targeting.

It is noteworthy that although Ly6g^low^ neutrophils possess potent CD8^+^ T cell-suppressive capacity, they accumulate later in the metastatic lungs. In contrast, Ly6g^high^ neutrophils infiltrate during the early establishment of pulmonary metastases. Consequently, inhibiting NETosis does not impair the immunosuppressive function of Ly6g^low^ neutrophils, as this subset fails to form NETs in response to stimuli such as IL-1β. Thus, NET-inhibitor therapy is likely only effective in alleviating the suppression mediated by Ly6g^high^ neutrophils, with no impact on the Ly6g^low^ subset. Therefore, combination strategies that concurrently target both neutrophil subsets warrant further investigation.

## Conclusion

Our study reveals that pulmonary neutrophils, classified as Ly6g^high^ and Ly6g^low^ subsets, are the predominant immune cell populations in the lung metastatic microenvironment. Ly6g^high^ neutrophils played an essential role in immunosuppression and immune evasion via NET-induced apoptosis of CD8^+^ T cells and reduction of infiltrating CD8^+^ T cells in the lung microenvironment. NET-derived cathelicidin is a key mediator for the role of Ly6g^high^ neutrophils by disrupting mitochondrial function in CD8^+^ T cells. Collectively, these findings highlight the importance of NETs and cathelicidin in BC lung metastasis, suggesting their potential as therapeutic targets for preventing metastatic progression.

## Ethical Approval

The study protocol was approved by the ethics committee of Chongqing Medical University (permit number: 2021084). The tissue samples were obtained with written consent from each patient. The animal study was carried out in compliance with the guidance suggestions of the Animal Care Committee of Chongqing Medical University (permit number: 2021084).

## Data Availability

The data and materials are available from the corresponding authors upon reasonable request. Bulk RNA-seq data for this study have been deposited in GEO under accession numbers GSE300115 and GSE300116 (https://www.ncbi.nlm.nih.gov/geo/). scRNA-seq data are accessible through the Genome Sequence Archive in the National Genomics Data Center, China National Center for Bioinformation/Beijing Institute of Genomics, Chinese Academy of Sciences (https://ngdc.cncb.ac.cn/gsa), with the accession number CRA014232.
