# Supplementary material for: The Ly6ghigh Neutrophil Subset Dictates Breast Cancer Lung Metastasis via CD8+ T Cell Death
Source: Cancer Commun (Lond). 2026 Jan 27;46:0003. doi: 10.34133/cancomm.0003 (PMC12857760; doi:10.34133/cancomm.0003)
Supplement: Supplementary 1 — Tables S1 to S4 Figs. S1 to S10 [file cancomm.0003.f1.zip › CANCOMM-D-25-00087.R3_Supplementary Materials_final.docx]

**Supplementary Materials for**

**The Ly6g^high^ neutrophil subset dictates breast cancer lung metastasis via CD8^+^ T cell death**

Rui Wang^1, 2^, Xiaoqi Liu^3^, Yixuan Hou^4^, Shanchun Chen^1^, Yongcan Liu^1^, Zexiu Lu^1^，Chao Chang^1^, Die Meng^1^, Jing Chen^1^, Xiaojiang Cui^5^, Zhengrong Shi^6, *^, Xueying Wan^1, *^, Manran Liu^1, 2, *^

**Supplementary Tables**

**Supplementary Table S1.** **Clinical characteristics and immunological profiling of healthy donors (*n* = 50).**

**Supplementary Table S2. Clinical characteristics and immunological profiling of breast cancer patients (*n* = 166).**

**Supplementary Table S3. Antibody information and sources.**

**Supplementary Table S4. Primer sequences for qPCR.**

**Supplementary Figures**


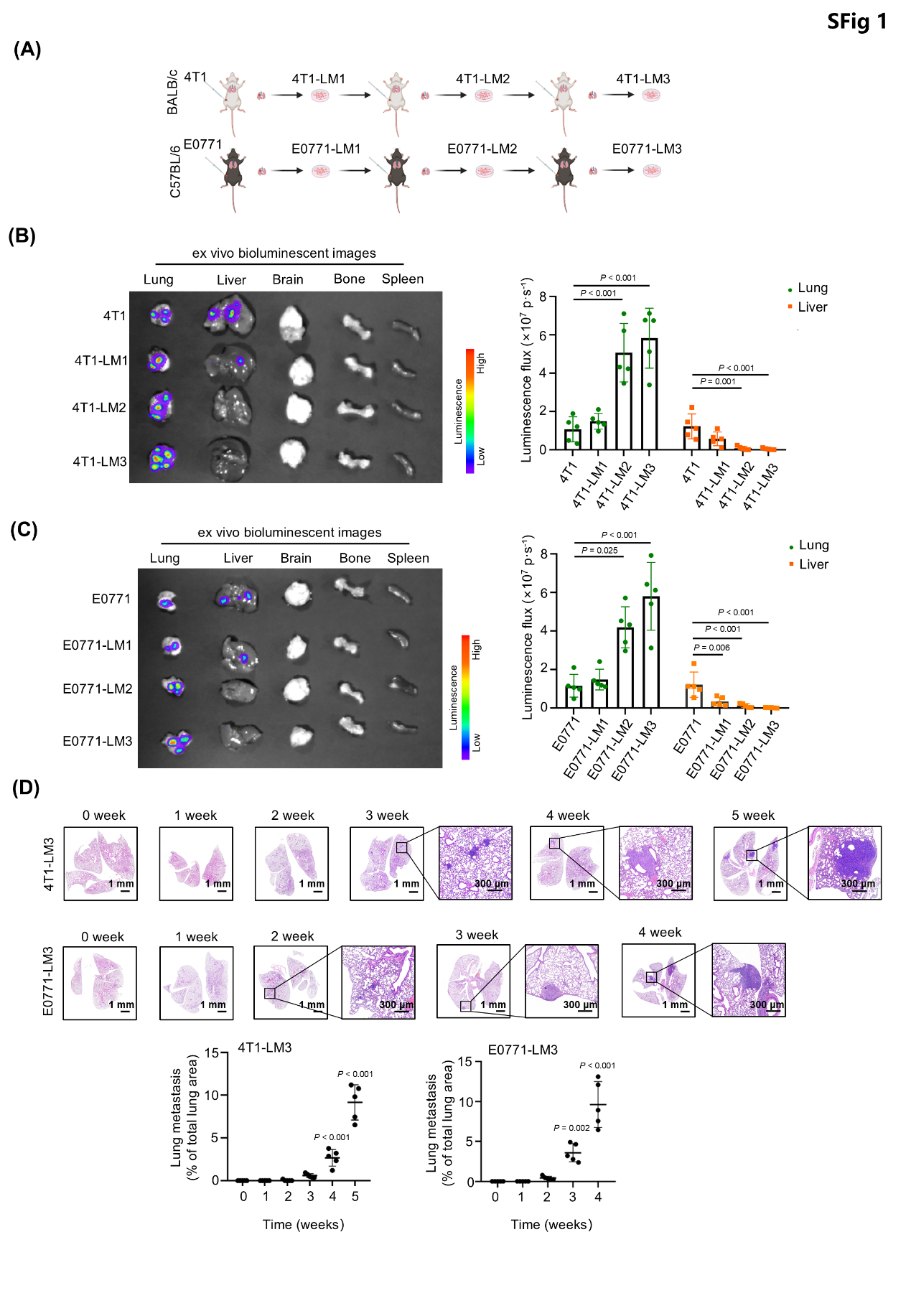


**Supplementary Figure S1. Generation of BC lung metastasis mouse models, related to Figure 1. (A)** Schematic overview of establishing BC lung metastasis mouse models. **(B)** Representative bioluminescence images show metastases of 4T1 cells in the major organs of tumor-bearing mice (*n* = 5). Quantification of bioluminescence intensity is shown in the bar graph on the right. Statistical significance was determined by comparison with mice injected with parental 4T1 cells. **(C)** Representative bioluminescence images showing metastases of E0771 cells in major organs of tumor-bearing mice (*n* = 5). Quantification of bioluminescence intensity is shown in the bar graph on the right. Statistical significance was determined by comparison with mice injected with parental E0771 cells. **(D)** 4T1-LM3 and E0771-LM3 BC cells were injected into the mouse mammary fat pads. Pulmonary metastases were examined by H&E staining weekly since cell injection, and the representative images are shown (*n* = 5). The data with error bars are presented as the mean ± SD; statistical significance was determined by two-way ANOVA (B-C) and one-way ANOVA test (D). Abbreviations: 4T1-LM, 4T1-lung metastasis; ANOVA, analysis of variance; BC, breast cancer; E0771-LM, E0771-lung metastasis; H&E, hematoxylin-eosin staining; SD, standard deviation.


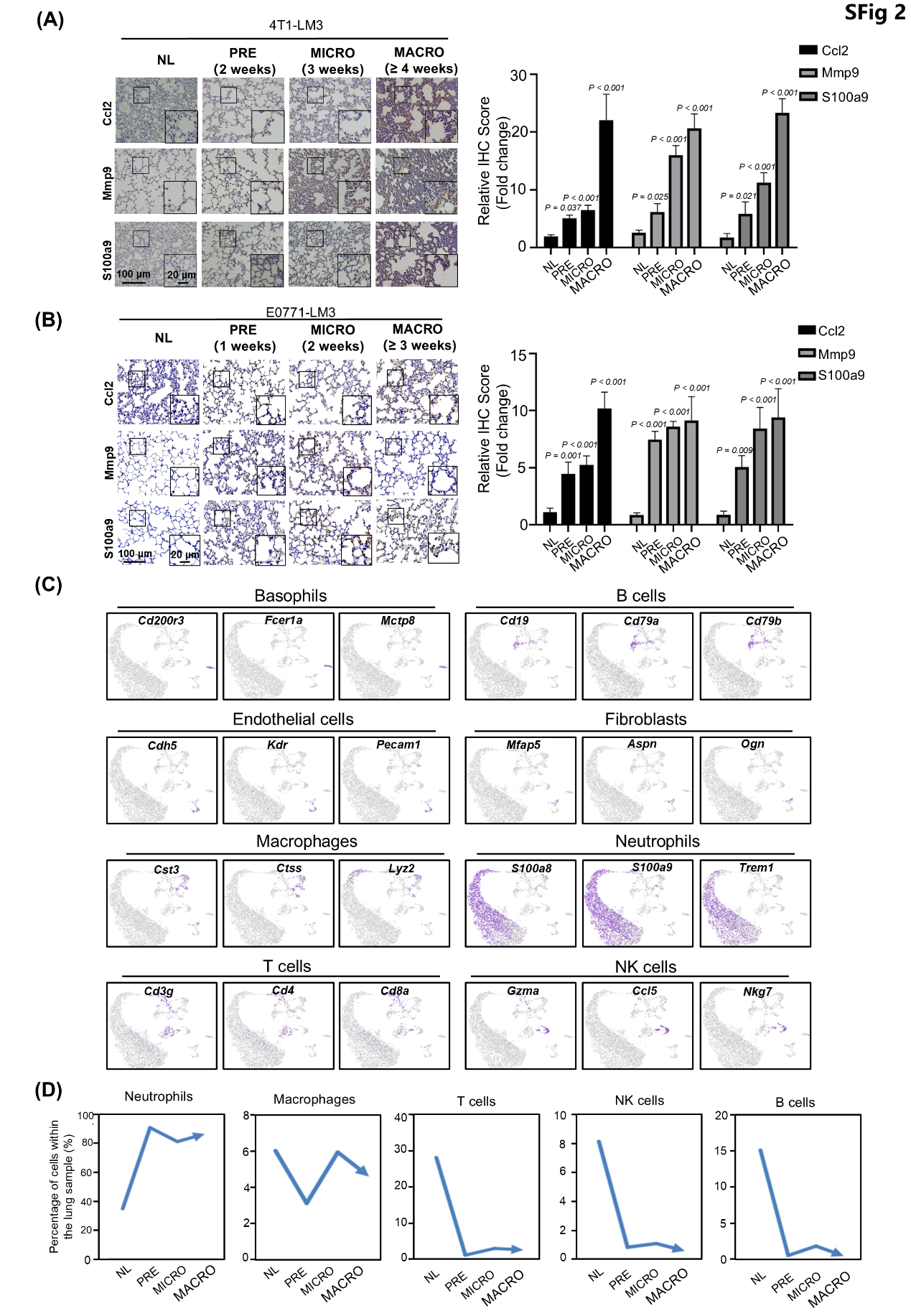


**Supplementary Figure S2. Identification of cell subpopulations through single-cell sequencing, related to Figure 1. (A-B)** Immunohistochemical staining for the pre-metastatic niche biomarkers Ccl2, Mmp9, and S100a9 in normal lung tissue and PRE, MICRO, and MACRO stages of lung metastasis. Representative lung tissue sections from mice [4T1-LM3 (BALB/c) model, *n* = 5, A; E0771-LM3 (C57BL/6) model, *n* = 5, B] are shown on the left. The quantitative analysis of staining intensity is shown in the bar graph on the right. **(C)** UMAP plots indicate the marker gene expression of the major cell lineages in lung tissues, as identified by scRNA-seq. **(D)** Dynamic changes in the abundance of immune cell populations during BC lung metastasis [4T1-LM3 (BALB/c) model]. The data with error bars are presented as the mean ± SD; statistical significance was determined by two-way ANOVA (A-B). Abbreviations: 4T1-LM3, 4T1-lung metastasis 3; *Aspn*, asporin; BC, breast cancer; Ccl2, c-c motif chemokine ligand 2; *Ccl5*, c-c motif chemokine ligand 5; *Cd19*, cluster of differentiation 19; *Cd200r3*, cluster of differentiation 200 receptor 3; *Cd3g*, cluster of differentiation cd3 gamma; *Cd4*, cluster of differentiation 4; *Cd79a*, cluster of differentiation 79 molecule; *Cd79b*, cluster of differentiation cd79b; *Cd8a*, cluster of differentiation 8a; *Cdh5*, cadherin 5; *Cst3*, cystatin c; *Ctss,* cathepsin s; E0771-LM3, E0771-lung metastasis 3; *Fcer1a*, fc fragment of ige receptor ia; *Gzma*, granzyme a; IHC, immunohistochemistry; *Kdr*, kinase insert domain receptor; *Lyz2*, lysozyme 2; MACRO, macro-metastatic lung; *Mctp8*, multiple c2 and transmembrane domain containing 8; *Mfap5*, microfibril associated protein 5; MICRO, micro-metastatic lung; Mmp9, matrix metallopeptidase 9; NK, natural killer; *Nkg7*, natural killer cell granule protein 7; NL, normal lung; *Ogn*, osteoglycin; *Pecam1*, platelet and endothelial cell adhesion molecule 1; PRE, pre-metastatic lung; *S100a8*, s100 calcium binding protein a8; *S100a9*, s100 calcium binding protein a9; S100a9, s100 calcium binding protein a9; scRNA-seq, single-cell RNA sequencing; SD, standard deviation; *Trem1*, triggering receptor expressed on myeloid cells 1; UMAP, uniform manifold approximation and projection.


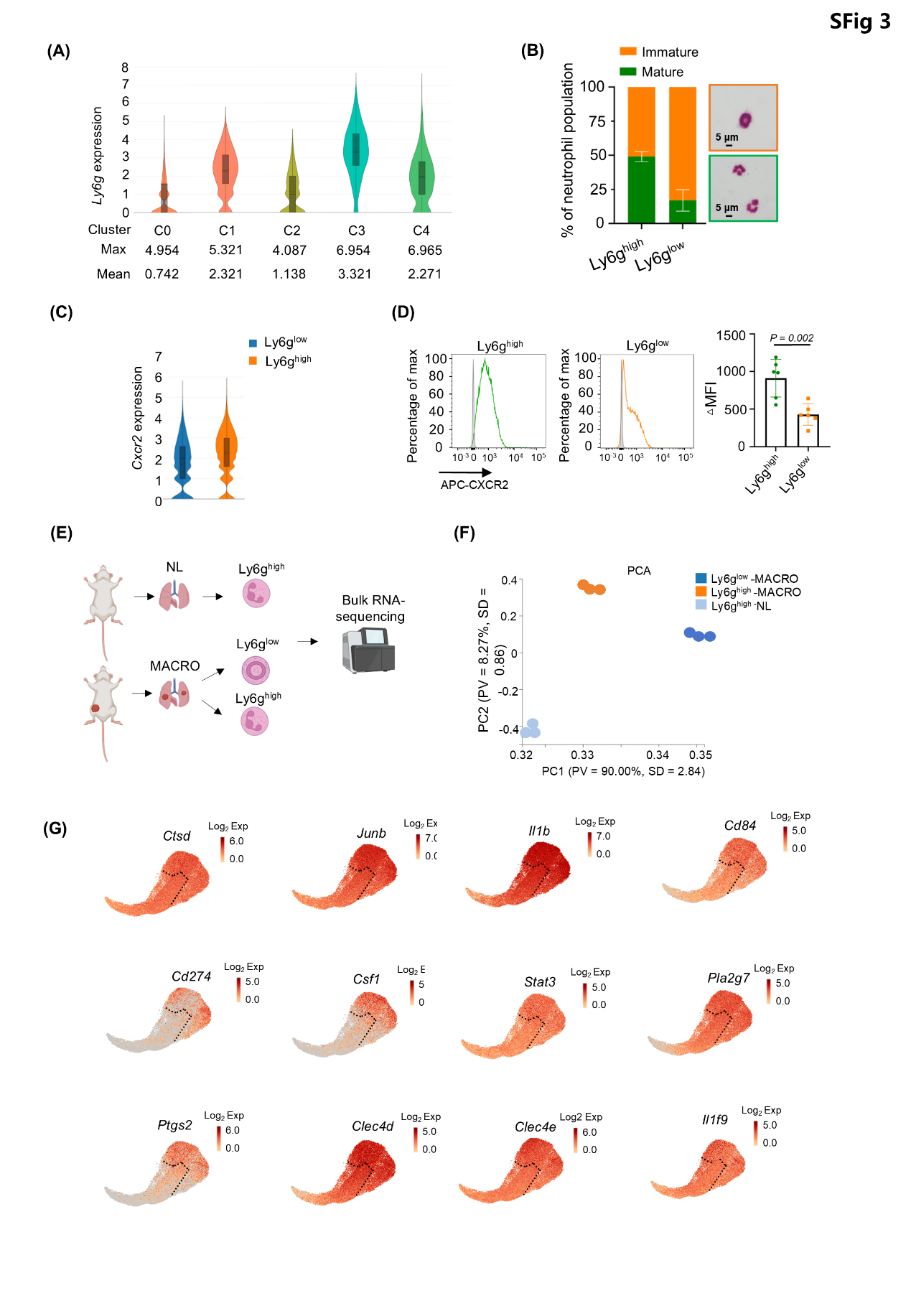


**Supplementary Figure S3. Identification of the Ly6g^high^ and Ly6g^low^** **neutrophil subsets in the lung metastasis microenvironment, related to Figure 2. (A)** Violin plots showing the expression of *Ly6g* in neutrophil clusters. **(B)** Representative images of Giemsa-stained Ly6g^high^ and Ly6g^low^ neutrophils showing the nuclear morphology of a ring-shaped immature nucleus (top) and a mature nucleus with clear nuclear segmentation (bottom). Nuclear morphology of Ly6g^high^ and Ly6g^low^ neutrophil subsets isolated from macro-metastases lung was scored to determine neutrophil maturity (*n* = 3). **(C)** Violin plots showing the expression of *Cxcr2* in Ly6g^high^ and Ly6g^low^ neutrophil subsets, as determined by scRNA-seq. **(D)** Cxcr2 expression levels in Ly6g^high^ and Ly6g^low^ neutrophils during the MACRO stage, as detected by flow cytometry [4T1-LM3 (BALB/c) model, *n* = 6]. **(E)** A schematic overview diagram shows that Ly6g^high^ neutrophils from normal lungs and Ly6g^high^ and Ly6g^low^ neutrophils from lung macro-metastases of tumor-bearing mice were isolated for bulk RNA-seq [4T1-LM3 (BALB/c) model]. **(F)** Principal component analysis of gene expression profiles in Ly6g^high^ neutrophils from normal lung tissue, and Ly6g^high^ and Ly6g^low^ neutrophils from lung macro-metastases (*n* = 3). **(G)** UMAP plots, colored based on expression of MDSC-related genes in neutrophils based on scRNA-seq. The dashed line separates the Ly6g^low^ (above) and Ly6g^high^ (below) neutrophil subsets. The data with error bars are presented as the mean ± SD; statistical significance was determined by Student’s *t*-test (D). Abbreviations: △MFI, the difference of mean fluorescence intensity; 4T1-LM3, 4T1-lung metastasis 3; APC, allophycocyanin; *Arg2*, arginase 2; *Cd274*, cluster of differentiation 274; *Cd84*, cluster of differentiation 84; *Clec4d*, c-type lectin domain family 4 member d; *Clec4e*, c-type lectin domain family 4 member e; *Csf1*, colony stimulating factor 1; *Ctsd*, cathepsin d; Cxcr2, c-x-c motif chemokine receptor 2; Exp, expression; *Il1b*, interleukin 1 beta; *Junb*, junb proto-oncogene; Ly6g, lymphocyte antigen 6 complex locus g; MACRO, macro-metastatic lung; MDSC, myeloid-derived suppressor cells; MFI, mean fluorescence intensity; NL, normal lung; *Ptgs2*, prostaglandin-endoperoxide synthase 2; PV, percent of variance; SD, standard deviation; scRNA-seq, single-cell RNA sequencing; *Stat3*, signal transducer and activator of transcription 3; UMAP, uniform manifold approximation and projection.


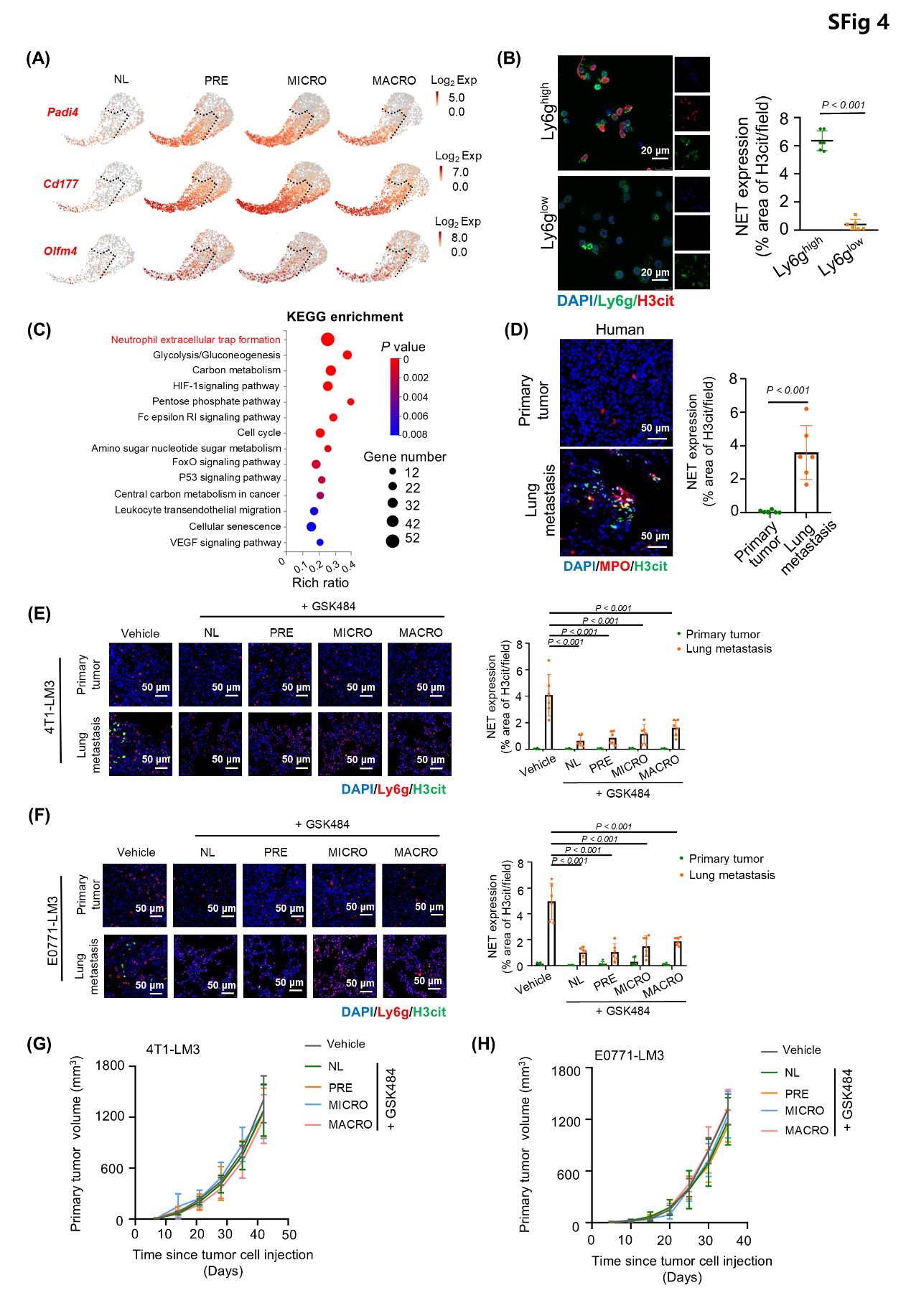


**Supplementary Figure S4. Ly6g^high^ neutrophils have a stronger NET formation ability, related to Figure 3. (A)** UMAP plots of neutrophils, color-coded for the expression of the genes *Padi4*, *Cd177,* and *Olfm4*. The dashed line separates the Ly6g^low^ (above) and Ly6g^high^ (below) neutrophil subsets. **(B)** Representative immunofluorescence micrographs showing NET formation of Ly6g^high^ and Ly6g^low^ neutrophils (*n* = 6) sorted by FACS and stimulated with PMA (200 nmol/L) for 3 hours *in vitro*. NETs were stained with antibodies against Ly6g (green) and H3cit (red), and nuclei were counterstained with DAPI (blue). The quantification of NET formation is shown in the bar graph on the right. **(C)** KEGG pathway enrichment analysis was performed using DEGs (absolute log_2_ fold change > 0.58) from bulk RNA-sequencing of Ly6g^high^ neutrophils in macro-metastatic versus normal lung tissues [4T1-LM3 (BALB/c) model]. **(D)** Representative immunofluorescence micrographs showing NETs formation in primary breast tumor tissues and pulmonary metastatic lesions from BC patients (*n* = 6). NETs were stained with antibodies against MPO (red) and H3cit (green), and nuclei were counterstained with DAPI (blue). The quantification of NET formation is shown in the bar graph on the right. **(E-F)** Representative immunofluorescence micrographs showing NETs formation in breast primary tumor and lung metastases from mice treated with or without PADI4 inhibitor (GSK484), with treatment initiation at different time points during metastasis [4T1-LM3 (BALB/c) model, *n* = 6, E; E0771-LM3 (C57BL/6) model, *n* = 6, F]. NETs were stained with antibodies against Ly6g (red) and H3cit (green), and nuclei were counterstained with DAPI (blue). The quantification of NET formation is shown in the bar graph on the right. **(G-H)** Tumor growth curves were measured [4T1-LM3 (BALB/c) model, *n* = 6, G; E0771-LM3 (C57BL/6) model, *n* = 6, H] following the same treatment regimen as indicated in (E-F). The data with error bars are presented as the mean ± SD; statistical significance was determined by Student’s *t*-test (B and D) and two-way ANOVA (E and F). Abbreviations: 4T1-LM3, 4T1-lung metastasis 3; *Cd177*, cluster of differentiation 177; DAPI, 4’,6-Diamidino-2-phenylindole; DEGs, differentially expressed genes; E0771-LM3, E0771-lung metastasis 3; Exp, expression; FACS, fluorescence-activated cell sorting; H3cit, citrullinated histone H3; KEGG, kyoto encyclopedia of genes and genomes; Ly6g, lymphocyte antigen 6 complex locus g; MACRO, macro-metastatic lung; MICRO, micro-metastatic lung; NETs, neutrophil extracellular traps; NL, normal lung; *Olfm4*, olfactomedin-4; *Padi4*, peptidyl arginine deiminase 4; PMA, phorbol-12-myristate-13-acetate; PRE, pre-metastatic lung; SD, standard deviation; UMAP, uniform manifold approximation and projection.


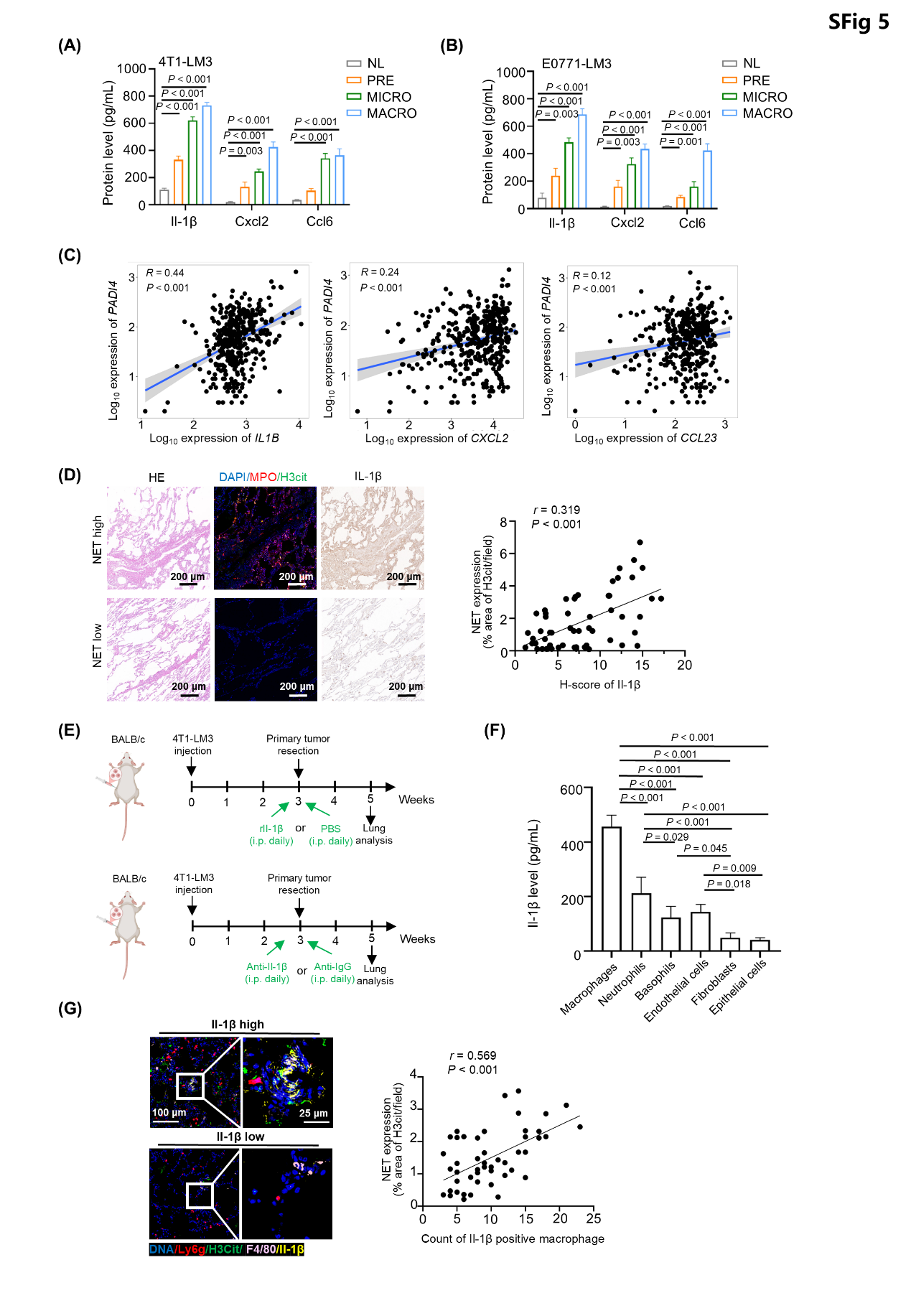


**Supplementary Figure S5. Il-1β induces NETosis in Ly6g^high^ neutrophil subsets in the lung metastasis niche, related to Figure 4. (A-B)** ELISA quantification of Il-1β, Cxcl2, and Ccl6 proteins in the lung tissues at different metastasis stages [4T1-LM3 (BALB/c) model, *n* = 3, A; E0771-LM3 (C57BL/6) model, *n* = 3, B]. **(C)** Correlation between *PADI4* and *IL1B*, *CXCL2*, and *CCL23* (named *Ccl6* in mouse) in human normal lung tissues (*n* = 391). The data were obtained from the TNM plot web server. **(D)** Representative H&E staining images, immunofluorescence images showing nuclei (blue), H3cit (green), and MPO (red), as well as immunohistochemical staining of IL-1β proteins in metastatic lesions from BC patients (*n* = 6). The correlation between IL-1β expression and NET formation was assessed by analyzing 10 randomly selected fields per patient. Representative immunofluorescence images of NET^high^ and NET^low^ neutrophils demonstrate their stratification according to H3cit staining intensity. **(E)** A schematic diagram of the treatment strategy for rIl-1β and anti-Il-1β antibodies. **(F)** ELISA quantification of Il-1β in conditioned medium from macrophages (CD11b^+^F4/80^+^), neutrophils (CD11b^+^Ly6g^+^), basophils (CD11b^+^CD200R3^+^), endothelial cells (CD45^-^CD31^+^), fibroblasts (CD45^-^CD31^-^CD326^-^), and epithelial cells (CD45^-^CD31^-^CD326^+^) separated from lungs at the macro-metastatic stage [4T1-LM3 (BALB/c) model; *n* = 4]. **(G)** Representative immunofluorescence images showing the relationship between Il-1β-positive macrophages and NETs in the lungs of the pre-metastasis stage [4T1-LM3 (BALB/c) model]. NETs were stained with antibodies against Ly6g (red) and H3cit (green), macrophages were stained with F4/80 (pink), and nuclei were counterstained with DAPI (blue). The correlation was assessed with randomly selected 10 view fields per mouse (*n* = 5). The data with error bars are presented as the mean ± SD; statistical significance was determined by two-way ANOVA (A and B), one-way ANOVA test (F), and the Pearson correlation analysis (D and G). Abbreviations: ANOVA, analysis of variance; BC, breast cancer; CCL23, c-c motif chemokine ligand 23; Ccl6, c-c motif ligand 6; Cxcl2, c-x-c motif chemokine ligand 2; DAPI, 4’,6-Diamidino-2-phenylindole; ELISA, enzyme-linked immunosorbent assay; H3cit, citrullinated histone H3; H&E, hematoxylin-eosin staining; Il-1β, interleukin-1 beta; IL-1β, interleukin-1 beta; i.p., intraperitoneal injection; Ly6g, lymphocyte antigen 6 complex locus g; MACRO, macro-metastatic lung; MICRO, micro-metastatic lung; MPO, myeloperoxidase; NL, normal lung; *PADI4*, peptidyl arginine deiminase 4; PRE, pre-metastatic lung; rIL-1β, recombinant interleukin-1 beta; SD, standard deviation.

**
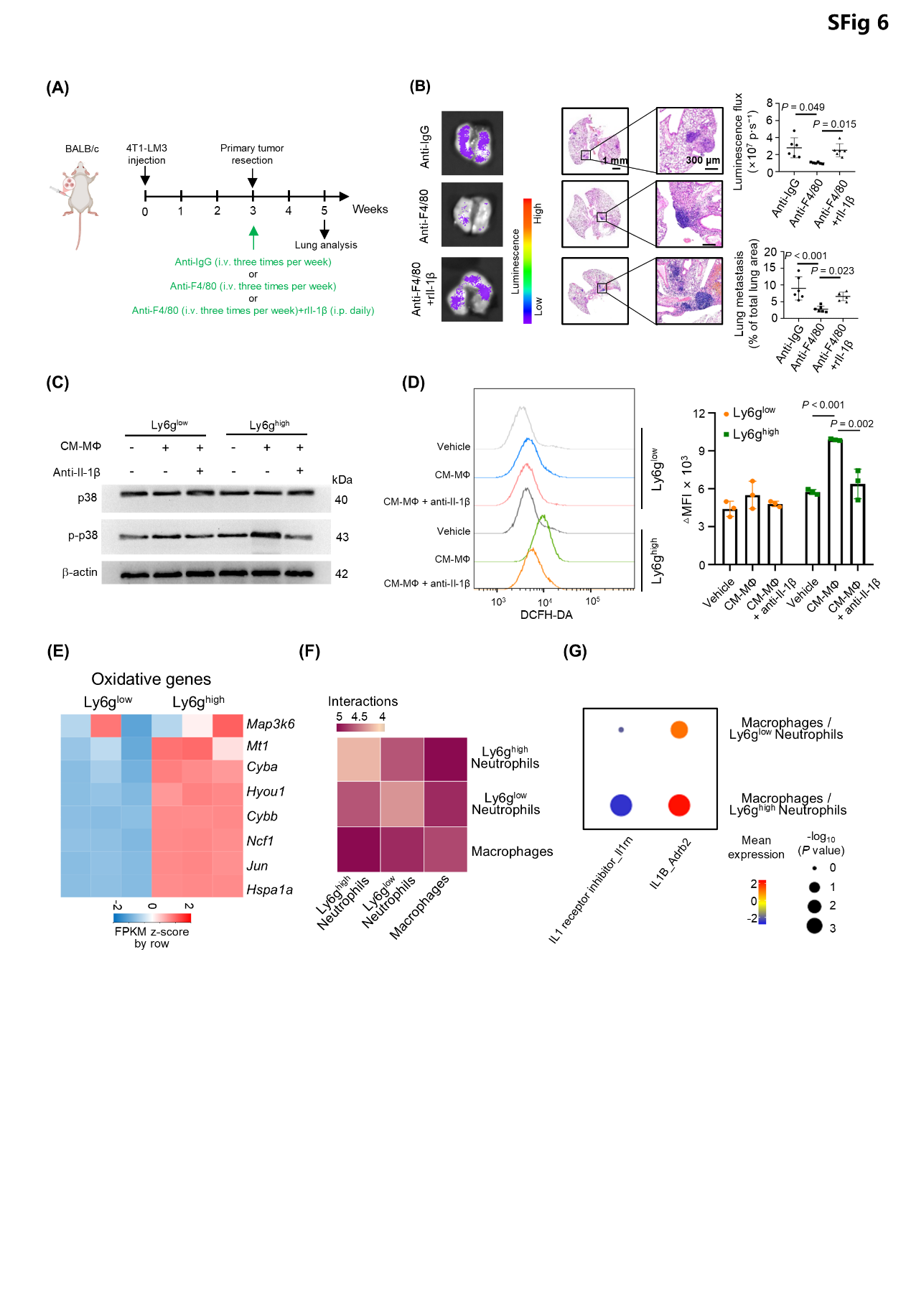
**

**Supplementary Figure S6. Macrophage-derived Il-1β induces NETosis in Ly6g^high^ neutrophils in the lung metastasis niche, related to Figure 4.** **(A)** Schematic illustration of the macrophage depletion. **(B)** Mice were treated with anti-IgG, an anti-F4/80 antibody alone, or anti-F4/80 antibody combined with rIl-1β, until the macro-metastatic stage [4T1-LM3 (BALB/c) model, *n* = 6]. Representative bioluminescence imaging and H&E staining images of the lung tissues were obtained at the macro-metastatic stage from the indicated groups. **(C-D)** FACS-sorted Ly6g^high^ and Ly6g^low^ neutrophils were pre-treated with vehicle (cell-free culture medium), CM-MΦ alone, or with anti-IL-1β antibody *in vitro* (*n* = 3). The protein expressions of p38 and phosphorylated p38 were evaluated by Western blotting; β-actin was utilized as an internal control (C). The ROS levels in Ly6g^high^ and Ly6g^low^ neutrophils were analyzed by FACS (D). **(E)** Heatmap displays the expression of oxidative genes in Ly6g^high^ and Ly6g^low^ neutrophils at the macro-metastatic stage based on the RNA-seq data (*n* = 3). **(F)** Cell-cell analysis interaction of Ly6g^high^ neutrophils, Ly6g^low^ neutrophils, and macrophages in the lungs, as analyzed via CellPhoneDB tools. **(G)** Dot plot showing the ligand-receptor interactions between macrophages and Ly6g^high^ or Ly6g^low^ neutrophils in the lung, as identified from scRNA-seq data using CellphoneDB. The data with error bars are presented as the mean ± SD; statistical significance was determined by a one-way ANOVA test (B and D). Abbreviations: △MFI, the difference of mean fluorescence intensity; 4T1-LM3, 4T1-lung metastasis 3; Adrb2, adrenoceptor beta 2; ANOVA, analysis of variance; MFI, mean fluorescence intensity; CM-MΦ, macrophage-derived conditioned medium; *Cyba*, cytochrome b-245 alpha chain; *Cybb*, cytochrome b-245 beta chain; DCFH-DA, 2',7'-Dichlorodihydrofluorescein diacetate; FACS, fluorescence-activated cell sorting; H&E, hematoxylin-eosin staining; *Hspa1a*, heat shock protein family a member 1a; *Hyou1*, hypoxia up-regulated 1; Il1rn, interleukin 1 receptor antagonist; Il-1β, interleukin-1 beta; i.p., intraperitoneal injection; i.v., intravenous; *Jun*, jun proto-oncogene; kDa, kilodalton; Ly6g, lymphocyte antigen 6 complex locus g*;* *Map3k6*, mitogen-activating protein kinase kinase 6; *Mt1*, metallothionein 1; *Ncf1*, neutrophil cytosolic factor 1; NETosis, neutrophil extracellular traps formation; rIl-1β, recombinant interleukin-1 beta; ROS, reactive oxygen species; scRNA-seq, single-cell RNA sequencing; SD, standard deviation.

**
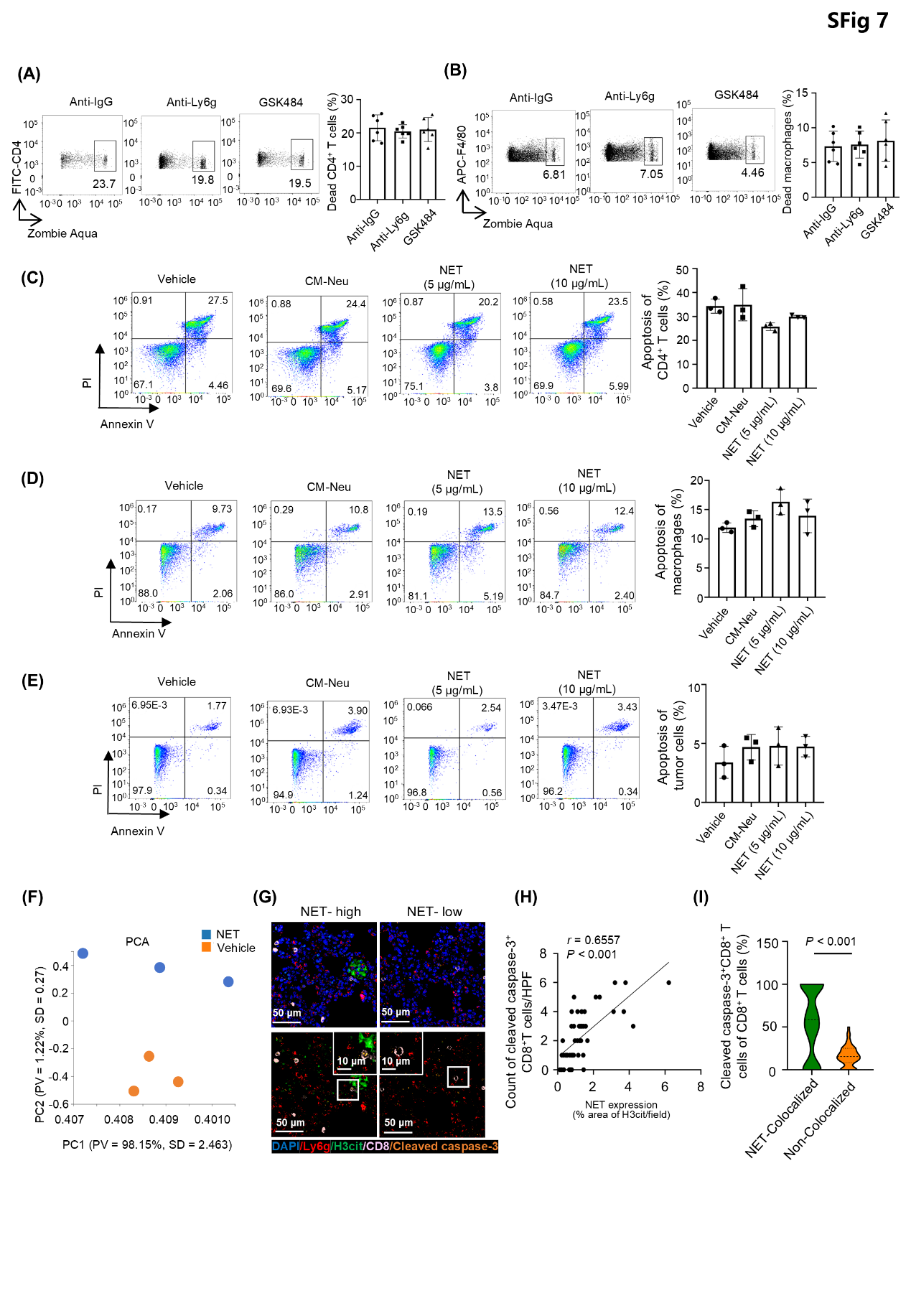
**

**Supplementary Figure S7. NETs induce CD8^+^ T cell apoptosis, related to Figure 5.**

**(A-B)** 4T1-LM3 (BALB/c) mouse model was treated with anti-IgG antibody, anti-Ly6g antibody, or PADI4 inhibitor (GSK484). CD4^+^ T cells (A) and macrophages (B) were isolated from the macro-metastatic lung and incubated with the cell death dye Zombie Aqua. The proportion of dead cells was analyzed by FACS. **(C-E)** CD4^+^ T cells (C, *n* = 3), macrophages (D, *n* = 3), and 4T1-LM3 cells (E, *n =* 3) were treated with vehicle (cell-free culture medium), CM-Neu, NETs (5 µg/mL), or NETs (10 µg/mL). The apoptotic cells were determined by FACS, and the corresponding quantification results were provided on the right. **(F)** PCA of gene expression in CD8^+^ T cells (*n* = 3) treated with or without NETs (10 μg/mL). **(G-H)** Representative immunofluorescence images showing the apoptotic CD8^+^ T cells spatially localized with NETs in the macro-metastatic lungs (G). The correlation was evaluated by randomly selecting 10 view fields per mouse (*n* = 5, H). Representative immunofluorescence images of NET^high^ and NET^low^ neutrophils demonstrate their stratification according to H3cit staining intensity. **(I)** Proportion of cleaved caspase-3⁺ CD8⁺ T cells located within NETs regions versus regions not colocalizing with NETs [4T1-LM3 (BALB/c) model, *n* = 5]. The data with error bars are presented as the mean ± SD; statistical significance was determined by one-way ANOVA test (A-E), Pearson correlation analysis in (H), and Student’s *t*-test (I). Abbreviations: 4T1-LM3, 4T1-lung metastasis 3; ANOVA, analysis of variance; APC, allophycocyanin; CD4, cluster of differentiation 4; CD8, cluster of differentiation 8; FACS, fluorescence-activated cell sorting; FITC, fluorescein isothiocyanate; Ly6g, lymphocyte antigen 6 complex locus g; NETs, neutrophil extracellular traps; CM-Neu, neutrophil-derived conditioned medium; PADI4, peptidylarginine deiminase 4; PCA, principal component analysis; PI, propidium iodide; PV, percent of variance; SD, standard deviation.

**
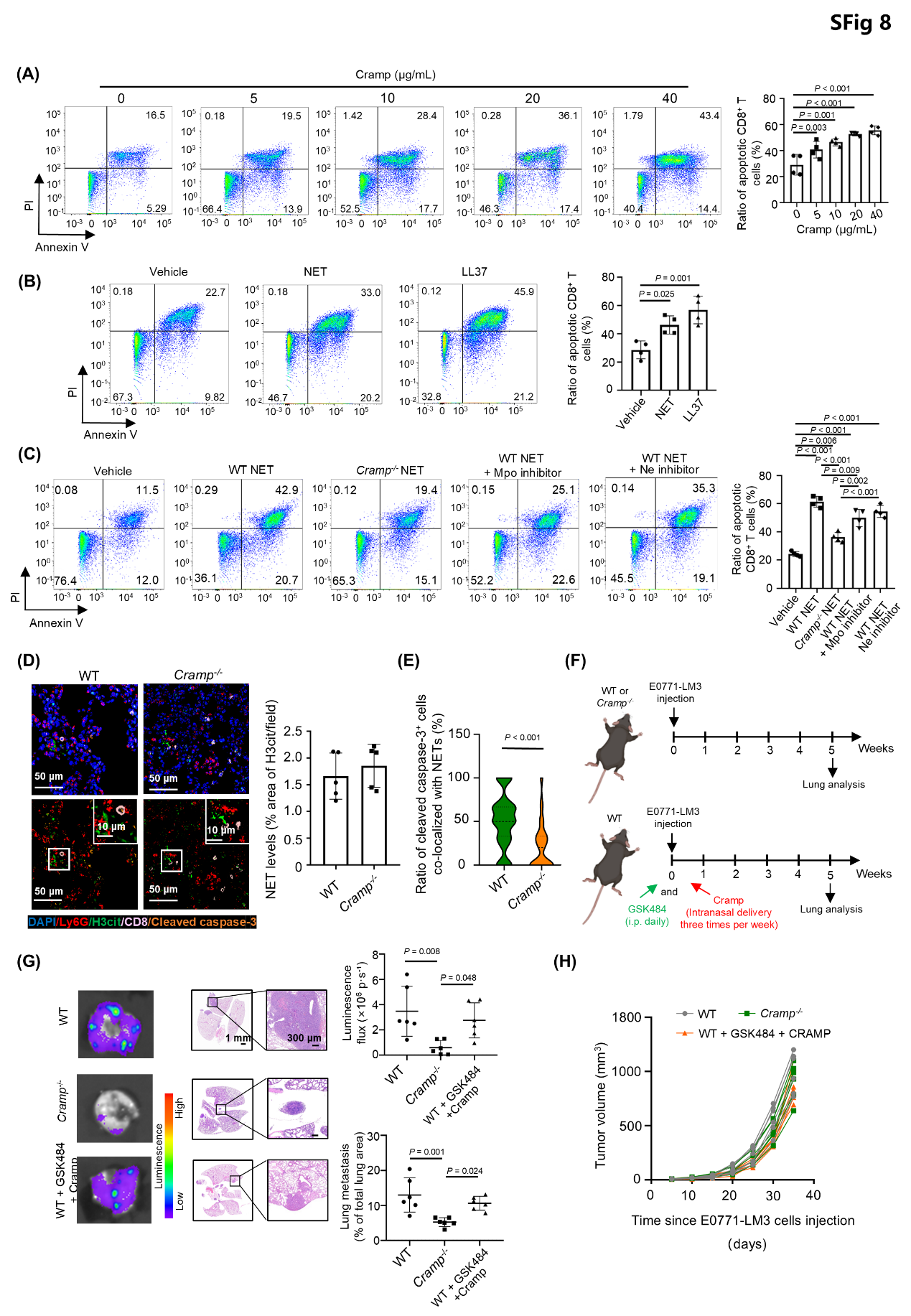
**

**Supplementary Figure S8. Reduced BC lung metastasis in *Cramp*^-/-^ mice, related to Figure 5. (A)** Mouse-derived CD8^+^ T cells were treated with different concentrations of Cramp. CD8^+^ T cell apoptosis was assessed by flow cytometry (*n* = 4). **(B)** Human-derived CD8^+^ T cells were treated with vehicle (cell-free culture medium), NETs (20 µg/mL), or LL37 (10 µg/mL). The apoptotic CD8^+^ T cells were quantified by flow cytometry (*n* = 4). **(C)** Mouse-derived CD8^+^ T cells were treated with vehicle (cell-free culture medium) WT NETs, *Cramp*^-/-^ NETs, NETs combined with Mpo inhibitor, or NETs combined with Ne inhibitor. Apoptotic cells were quantified by flow cytometry (*n* = 4). **(D)** Representative immunofluorescence images showing the apoptotic CD8^+^ T cells spatially localized with NETs in the pre-metastatic lung of WT mice or *Cramp^-/-^* mice injected with E0771-LM3 cells (*n* = 6). **(E)** The proportion of apoptotic CD8^+^ T cells within NET-rich areas in pre-metastatic lung from WT mice or *Cramp^-/-^* mice injected with E0771-LM3 cells. **(F-H)** WT or *Cramp^-/-^* mice were injected with E0771-LM3 cells. WT mice were subsequently treated with PADI4 inhibitor (GSK484) and Cramp (*n* = 6). A schematic illustrates the experiment design (F). Representative bioluminescence imaging and H&E staining images of the lung tissues at the macro-metastatic stage from the indicated groups (G), and the tumor growth curves of each group were provided (H). The data with error bars are presented as the mean ± SD; statistical significance was determined by one-way ANOVA test (A-C, and G), and Student’s *t*-test (D). Abbreviations: ANOVA, analysis of variance; BC, breast cancer; CD8, cluster of differentiation 8; Cramp, cathelicidin antimicrobial peptide; E0771-LM3, E0771-lung metastasis 3; H&E, hematoxylin-eosin staining; i.p., intraperitoneal injection; Ly6g, lymphocyte antigen 6 complex locus g; Mpo, myeloperoxidase; Ne, neutrophil elastase; NETs, neutrophil extracellular traps; PADI4, peptidylarginine deiminase 4; PI, propidium iodide; WT, wild type; SD, standard deviation.

**
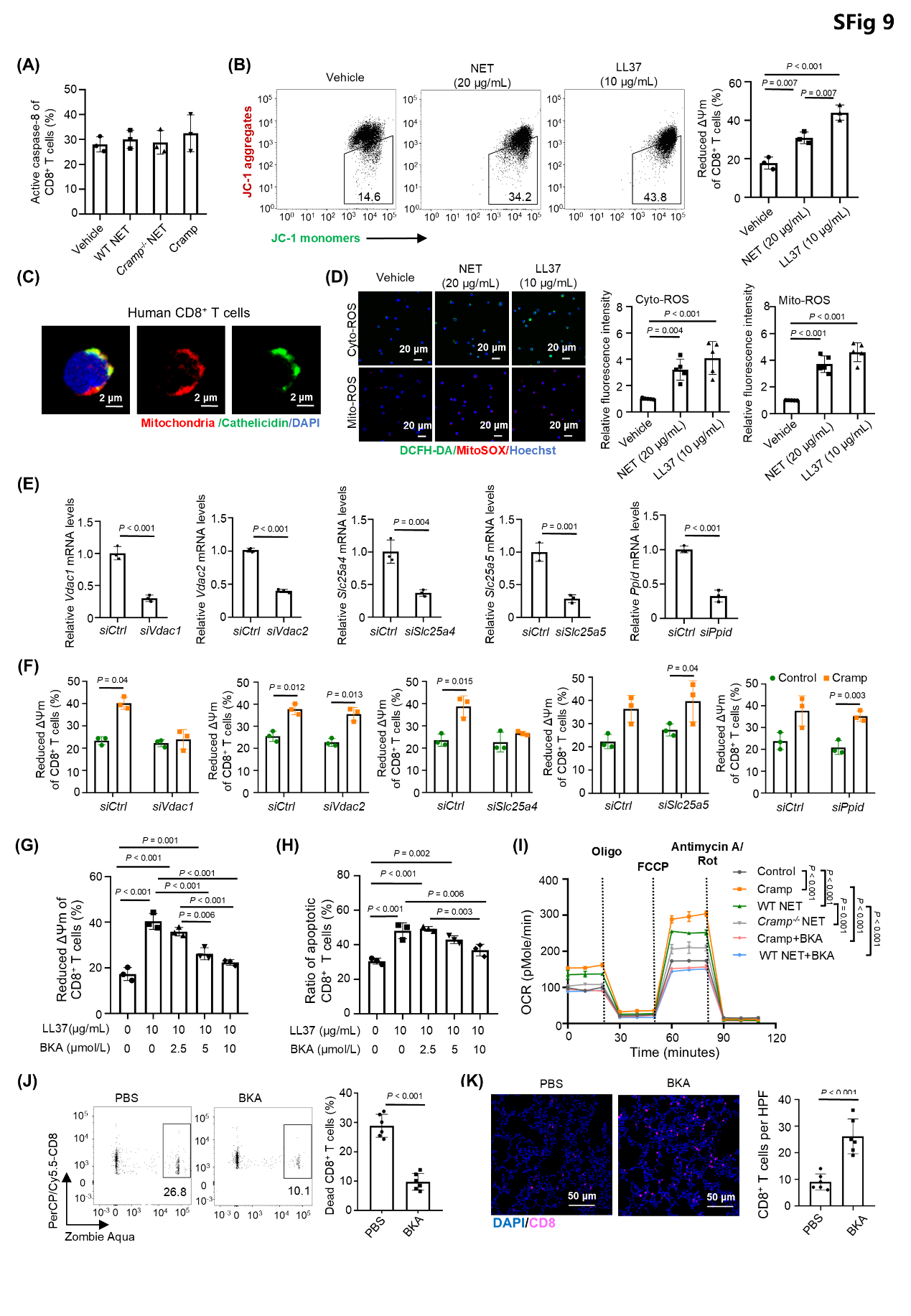
**

**Supplementary Figure S9. Cathelicidin binding to Ant1 induces mPTP opening and promotes cell apoptosis in human CD8^+^ T cells, related to Figure 6. (A)** CD8^+^ T cells were isolated from WT mice and subsequently stimulated with vehicle (cell-free culture medium), NETs (10 µg/mL) derived from WT or *Cramp*^-/-^ mice, or Cramp (20 µg/mL). The expression levels of caspase-8 were then analyzed by flow cytometry (*n* = 3). **(B)** CD8^+^ T cells derived from human peripheral blood were treated with vehicle (cell-free culture medium), NETs (20 µg/mL) derived from stimulated human peripheral blood-derived neutrophils, or LL37 (10 µg/mL) and subsequently stained with JC-1 (*n* = 3). ΔΨm was determined by flow cytometry. JC-1 monomers and aggregates were stained as green and red, respectively. **(C)** Representative immunofluorescence images showing co-localization of NET-derived cathelicidin (green) with the mitochondria (red) in human CD8^+^ T cells examined by confocal laser microscopy. **(D)** Human CD8^+^ T cells were treated with NETs or LL37. Cytoplasmic and mitochondrial ROS were measured using DCFH-DA and MitoSOX Red probes (*n* = 5; Scale bars: 20 µm). **(E)** Knockdown of *Vdac1*, *Vdac2*, *Slc25a4*, *Slc25a5*, and *Ppid* was performed in CD8⁺ T cells isolated from WT mice and the efficiency was determined by qPCR (*n* = 3). **(F)** The *Vdac1-*, *Vdac2-*, *Slc25a4-*, *Slc25a5-,* or *Ppid-*deficient CD8^+^ T cells were treated with Cramp (20 µg/mL), and then the ΔΨm of CD8^+^ T cells was assessed by JC-1 staining (*n* =3). **(G-H)** Human CD8^+^ T cells were treated with LL37 and different concentrations of BKA. ΔΨm by JC-1 staining (G) and cell apoptosis (H) were assessed using flow cytometry (*n* = 3). **(I)** CD8⁺ T cells were treated with Cramp (20 µg/mL), WT NETs (10 µg/mL), *Cramp^-/-^* NETs (10 µg/mL), a combination of Cramp (20 µg/mL) or WT NETs (10 µg/mL) with BKA (10 μmol/L). The oxidative phosphorylation-related metrics were tested (*n* =3). **(J-K)** 4T1-LM3 (BALB/c) mice model was administered PBS or BKA (200 μg/kg) three times weekly. CD8^+^ T cells isolated from the macro-metastatic lungs of BALB/c mice were incubated with cell death dye Zombie Aqua and T cell surface antibodies. The proportion of dead CD8^+^ T cells was evaluated by flow cytometry (*n* = 6, J). Representative immunofluorescence staining and quantitation of CD8^+^ T cells in macro-metastasis lungs are provided (K). All the data with error bars are presented as the mean ± SD; statistical significance was determined by one-way ANOVA test (A, B, D, G, H, and I), two-way ANOVA test (F), and Student’s *t*-test (E, J, and K). Abbreviations: ANT1, adenine nucleotide translocator 1; ANOVA, analysis of variance; BKA, bongkrekic acid; CD8, cluster of differentiation 8; Cramp, cathelicidin antimicrobial peptide; DCFH-DA, 2',7'-dichlorodihydrofluorescein diacetate; FCCP, carbonyl cyanide 4 phenylhydrazone; mPTP, mitochondrial permeability transition pore; NETs, neutrophil extracellular traps; *Ppid*, peptidylprolyl isomerase d; qPCR, quantitative real-time polymerase chain reaction; ROS, reactive oxygen species; Rot, rotenone; *Slc25a4*, solute carrier family 25 member 4; *Slc25a5*, solute carrier family 25 member 5; *Vdac1*, voltage-dependent anion channel 1; *Vdac2*, voltage-dependent anion channel 2; WT, wild type; SD, standard deviation; ΔΨm, mitochondrial membrane potential.


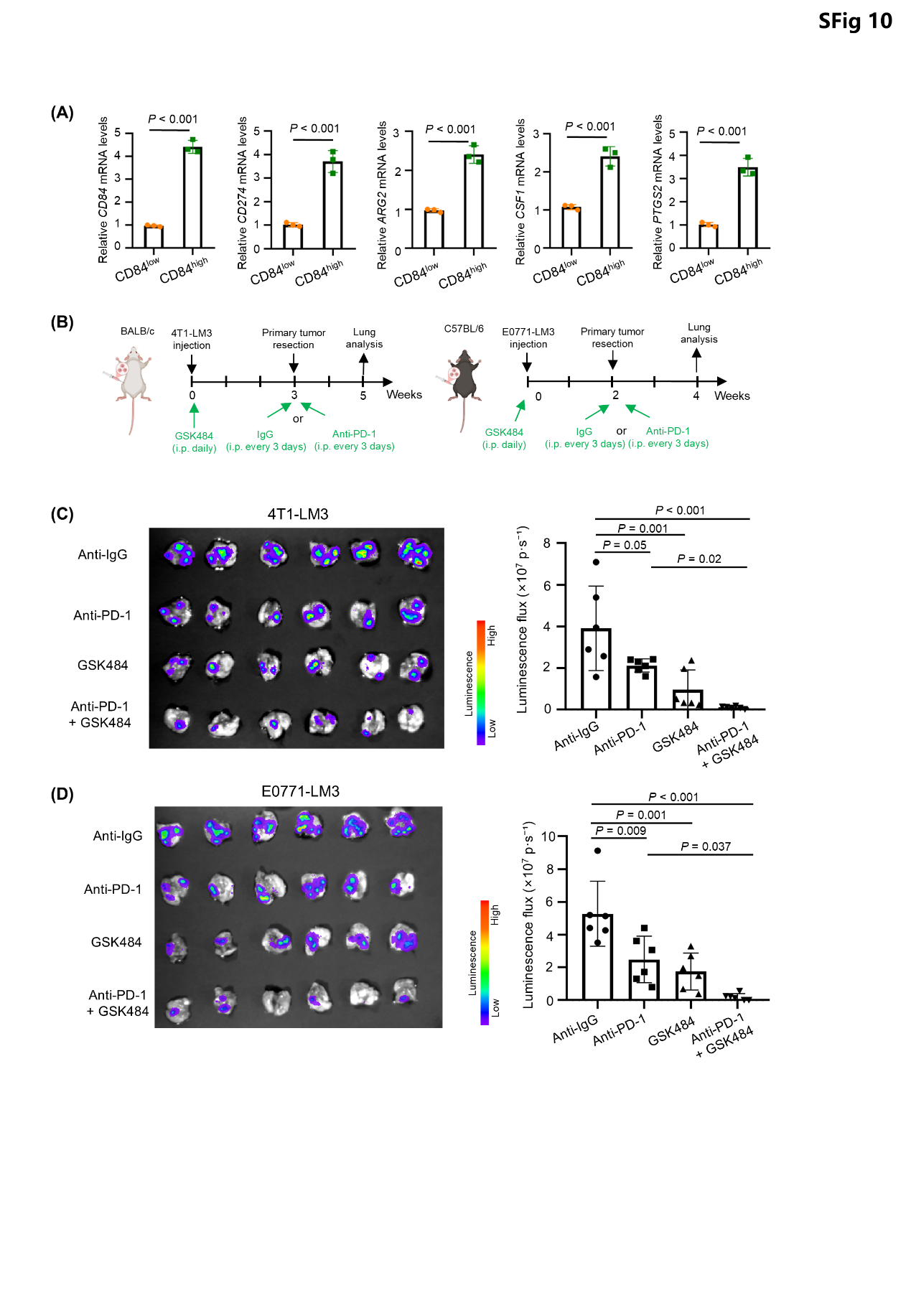


**Supplementary Figure S10. CD84^high^ neutrophils possess MDSC characteristics, related to Figure 7. (A)** The mRNA expression levels of *CD84*, *CD274*, *ARG2*, *CSF1*, and *PTGS2* in CD84^high^ and CD84^low^ neutrophils isolated from the peripheral blood of BC patients with pulmonary metastases were quantified by qPCR (*n* = 3). **(B)** Schematic illustration of the treatment strategy with anti-IgG, anti-PD-1 antibodies, PADI4 inhibitor (GSK484), or anti-PD-1 antibodies combined with PADI4 inhibitor. **(C-D)** Representative bioluminescence images of lung metastases in mice at the macro-metastatic stages treated with anti-IgG (200 μg/mouse), anti-PD-1 antibodies (200 μg/mouse), PADI4 inhibitor (GSK484), or anti-PD-1 antibodies combined with GSK484 (*n* = 6). The bar graph on the right quantifies the lung luminescence. The data with error bars are presented as the mean ± SD; statistical significance was determined by Student’s *t*-test (A) and one-way ANOVA test (C and D). Abbreviations: 4T1-LM3, 4T1-lung metastasis 3; *ARG2*, Arginase 2; CD84, cluster of differentiation 84; *CD274,* cluster of differentiation 274*; CSF1*, colony-stimulating factor-1; E0771-LM3, E0771-lung metastasis 3; MDSC, myeloid-derived suppressor cell; *PTGS2*, prostaglandin-endoperoxide synthase 2; qPCR; quantitative real-time polymerase chain reaction; SD, standard deviation.
